# The β2-adrenergic receptor in the apical membrane of intestinal enterocytes senses sugars to stimulate glucose uptake from the gut

**DOI:** 10.3389/fcell.2022.1041930

**Published:** 2023-01-09

**Authors:** Frederik Paulussen, Chetan P. Kulkarni, Frank Stolz, Eveline Lescrinier, Stijn De Graeve, Suzan Lambin, Arnaud Marchand, Patrick Chaltin, Peter In't Veld, Joseph Mebis, Jan Tavernier, Patrick Van Dijck, Walter Luyten, Johan M. Thevelein

**Affiliations:** ^1^ Center for Microbiology, VIB, Leuven-Heverlee, Belgium; ^2^ Laboratory of Molecular Cell Biology, Institute of Botany and Microbiology, KU Leuven, Leuven-Heverlee, Belgium; ^3^ Functional Genomics and Proteomics Research Unit, Department of Biology, KU Leuven, Leuven, Belgium; ^4^ Medicinal Chemistry, Rega Institute for Medical Research, KU Leuven, Leuven, Belgium; ^5^ Leuven Research & Development, Leuven, Belgium; ^6^ Department of Pathology, Free University of Brussels, Brussels, Belgium; ^7^ Department of Pathology, KU Leuven, Flanders, Belgium; ^8^ Department of Biochemistry, Faculty of Medicine and Health Sciences, Ghent University, Ghent, Belgium; ^9^ Center for Medical Biotechnology, VIB, Ghent, Belgium; ^10^ NovelYeast bv, Bio-Incubator BIO4, Gaston Geenslaan 3, Leuven-Heverlee,, Belgium

**Keywords:** glucose sensing, gut, enterocytes, Beta2-adrenergic receptor, glucose transport, SGLT1, beta2-adrenergic receptor antagonists

## Abstract

The presence of sugar in the gut causes induction of SGLT1, the sodium/glucose cotransporter in intestinal epithelial cells (enterocytes), and this is accompanied by stimulation of sugar absorption. Sugar sensing was suggested to involve a G-protein coupled receptor and cAMP - protein kinase A signalling, but the sugar receptor has remained unknown. We show strong expression and co-localization with SGLT1 of the β2-adrenergic receptor (*β*_2_-AR) at the enterocyte apical membrane and reveal its role in stimulating glucose uptake from the gut by the sodium/glucose-linked transporter, SGLT1. Upon heterologous expression in different reporter systems, the *β*_2_-AR responds to multiple sugars in the mM range, consistent with estimated gut sugar levels after a meal. Most adrenergic receptor antagonists inhibit sugar signaling, while some differentially inhibit epinephrine and sugar responses. However, sugars did not inhibit binding of I^125^-cyanopindolol, a *β*_2_-AR antagonist, to the ligand-binding site in cell-free membrane preparations. This suggests different but interdependent binding sites. Glucose uptake into everted sacs from rat intestine was stimulated by epinephrine and sugars in a *β*_2_-AR-dependent manner. STD-NMR confirmed direct physical binding of glucose to the *β*_2_-AR. Oral administration of glucose with a non-bioavailable *β*_2_-AR antagonist lowered the subsequent increase in blood glucose levels, confirming a role for enterocyte apical *β*_2_-ARs in stimulating gut glucose uptake, and suggesting enterocyte *β*_2_-AR as novel drug target in diabetic and obese patients. Future work will have to reveal how glucose sensing by enterocytes and neuroendocrine cells is connected, and whether *β*_2_-ARs mediate glucose sensing also in other tissues.

## Introduction

Diabetes and obesity are two widespread diseases that could strongly profit from a precisely controlled reduction of sugar uptake from the gut. Extensive evidence has been reported that the main cells in the intestinal epithelium, the enterocytes, have mechanisms to sense the presence of sugar in the gut and respond by increasing sugar uptake from the gut lumen (Dockray, 2003). Most of the glucose uptake, at least at lower intestinal glucose concentrations, is mediated by the Na^+^-glucose linked transporter SGLT1, located in the apical membrane of enterocytes facing the intestinal lumen (Ferraris, 2001). At higher intestinal glucose concentrations, recruitment of GLUT2 from the basolateral to the apical membrane might support further increases in glucose uptake (Kellett and Helliwell, 2000; Gromova et al., 2021). Sugar-induced upregulation of SGLT1 activity and corresponding stimulation of glucose uptake from the gut is well-documented (Diamond and Karasov, 1987; Sharp et al., 1996; Dyer et al., 2003; Shirazi-Beechey et al., 2011a). A wide range of sugars, including non-permeable sugar analogs, is able to elicit the response, which requires no metabolism of transported sugars (Miyamoto et al., 1993). Evidence was obtained that the sugar sensing involved a G protein-coupled receptor (GPCR) acting through the cAMP-Protein Kinase A (PKA) pathway (Sharp and Debnam, 1994; Dyer et al., 2003; Shirazi-Beechey et al., 2011a). Sweet taste receptors were shown to be involved in sugar-induced stimulation of glucose uptake by enterocytes (Dyer et al., 2005; Margolskee et al., 2007) and carnivores lacking a functional sweet taste receptor, like cats, also lack glucose-induced stimulation of glucose transport in response to dietary sugar (Buddington et al., 1991; Batchelor et al., 2011). Later work revealed that expression of the sweet taste receptors is confined to certain intestinal neuroendocrine cells (representing less than 1% of intestinal epithelial cells) to regulate the release of incretin peptide hormones that somehow stimulate glucose uptake in the absorptive enterocytes (Sternini et al., 2008; Steinert and Beglinger, 2011). How these peptide hormones control SGLT1 activity in the neighbouring enterocytes has remained unclear (Glendinning, 2018). Over 99% of intestinal epithelial cells are absorptive enterocytes that express SGLT1, whose activity is regulated by the glucose level in the gut (Dyer et al., 2003). The increase in enterocyte glucose transport starts within seconds of exposure to sugars, which appears too fast for the proposed neuroendocrine loop (Dyer et al., 2005), but rather suggests an additional cell-autonomous process in the enterocyte itself. Moreover, incretin secretion is also stimulated by lipids and proteins (Ezcurra et al., 2013), which do not trigger increased glucose transport, suggesting an additional requirement for sugar regulation of SGLT1. Thus, there seems to exist an elusive sugar-specific sensor in the enterocytes regulating sugar uptake *via* control of SGLT1 activity.

We have previously discovered a glucose/sucrose-sensing GPCR, Gpr1, in the yeast *Saccharomyces cerevisiae*, which was the first nutrient-sensing GPCR discovered. Gpr1 regulates energy and reserve carbohydrate metabolism through the cAMP-PKA signaling pathway in response to glucose and other rapidly-fermented sugars (Kraakman et al., 1999; Lemaire et al., 2004). Similar sugar-sensing receptors belonging to the same GPCR subfamily and also acting through the cAMP-PKA pathway have been found in *Schizosaccharomyces pombe* (Welton and Hoffman, 2000), *Candida albicans* (Miwa et al., 2004; Maidan et al., 2005), *Neurospora crassa* (Li and Borkovich, 2006), and homologs are present in many other fungi (Xue et al., 2008). The physiological function of the GPCR Gpr1 in *S. cerevisiae* resembles the regulation of energy and reserve metabolism in certain mammalian cell types, e.g. liver and fat cells, by the β2-adrenergic receptor (*β*_2_-AR) in response to epinephrine (Arner, 2005; Rui, 2014). The *β*_2_-AR is a major drug target and likely the best-characterized mammalian GPCR (Frishman, 2008; Shukla et al., 2008). Its three-dimensional structure has been determined for different agonist- and G_s_-protein-associated states (Cherezov et al., 2007; Rasmussen et al., 2007; Rosenbaum et al., 2007; Rasmussen et al., 2011a; Rasmussen et al., 2011b). The *β*_2_-AR belongs to a family of closely related adrenergic receptors responsive to catecholamines, like epinephrine and norepinephrine (Strosberg, 1993). Antagonists and agonists with varying specificity for the different α- and β-adrenergic receptor subtypes have been identified, and many of those are in clinical use as drugs, e.g. in cardiovascular and respiratory medicine (Smith and Teitler, 1999; Baker, 2005; Tyndall and Sandilya, 2005; Frishman, 2008).

Following our discovery of the yeast sugar-sensing GPCR Gpr1, we have explored the possible presence of a sugar-sensing GPCR in the apical membrane of mammalian enterocytes. Both cell types are exposed to highly variable nutrient conditions in their environment, and both also respond to glucose with specific GPCR- and cAMP/PKA-dependent responses controlling carbohydrate metabolism. As shown in the present paper, we surprisingly found that the *β*_2_-AR is abundantly present in the apical membrane of enterocytes, a location more suggestive of a function as sensor for components like nutrients in the gut lumen, rather than for catecholamine sensing. The use of two different heterologous expression systems subsequently revealed that the *β*_2_-AR responds to multiple sugars at concentrations (5–100 mM) found in the gut after a meal. Experiments with everted sacs prepared from rat intestine, and determination of blood glucose levels after *in vivo* administration of glucose together with a non-bioavailable *β*-AR antagonist confirmed that the *β*_2_-AR mediates sugar-induced stimulation of glucose uptake from the gut lumen. The confirmation by STD-NMR of direct binding of glucose at mM concentrations to the *β*_2_-AR, hints at a possible more universal role of the *β*_2_-AR as glucose-sensing receptor in other mammalian cell types. Our results suggest that the *β*_2_-AR evolved from an ancient sugar receptor in an ancestral primitive unicellular eukaryote, and has retained its sugar-binding capacity to function as a sugar-sensor in the gut and possibly in other mammalian tissues.

## Materials and methods

### PCR techniques

The templates used were RNA extracts from mouse mucosal scrapings and from STC-1 cells, isolated at the laboratory of Prof. Shirazi-Beechey (University of Liverpool), sent on dry ice and stored at −80°C. First-strand cDNA synthesis was achieved using the RevertAid™ H Minus First Strand cDNA synthesis kit (Fermentas) according to the manufacturer’s protocol, using 5 µg of purified RNA. Both oligo(dT) and random hexamer primers were used in the reaction. The resulting cDNA was stored at −20°C. Alternatively, the Thermoscript^®^ RT kit (Invitrogen) was used for cDNA synthesis according to the supplied protocol. PCR primers are listed in Supporting Information Table S1.

Quantitative PCR analysis was performed with SYBR^®^ Green. Reactions were initiated with 100 ng of cDNA, 300 ng of both primers and the SYBR green MasterMix (Eurogentec) in a total reaction volume of 25 µl. Between 40–50 amplification cycles were performed in the ABI Prism^®^ 7000 apparatus (Applied Biosystems).

### Microarray genome-wide gene expression analysis

RNA from STC-1 cells, cultured either in the presence of 5 or 25 mM glucose for the last 24 h, was used as template. First-strand cDNA synthesis was achieved using the RevertAid™ H Minus First Strand cDNA synthesis kit (Fermentas) according to the supplied protocol, using 5 µg of purified RNA. Both oligo(dT) and random hexamer primers were used in the reaction. The resulting cDNA was cleaned using the QIAquick PCR Purification Kit (Qiagen), and was used by the VIB Microarray Facility (VIB MAF vzw, Leuven, Belgium) for whole-genome expression analysis with Affymetrix GeneChip^®^ Mouse Genome 430 2.0 Arrays.

### Immunohistochemistry

Mouse and human tissues were embedded in paraffin after formaldehyde fixation. The human tissue used was from archived patient biopsies embedded in paraffin. Both tissue samples were taken from the duodenal part of the small intestine. Anti-hu*β*_2_-AR (1/100) (Abcam) was used as primary antibody for peroxidase staining on human tissue. We also detected *β*_2_-AR in control tissues known to express this receptor using the same antibodies, supporting antibody specificity. Secondary antibodies were peroxidase-labeled goat anti-rabbit antibodies (Abcam). The stained tissues were developed with 3,3′-diaminobenzidine, and they were counterstained with hematoxylin (Gill III). Antibodies used for fluorescent staining and co-localization in mouse tissue were rabbit anti- *β*_2_-AR (Assay Designs) (1/50) and goat anti-SGLT1 (M19, Santa Cruz) (1/50). Secondary antibodies used were anti-rabbit immunoglobulin labeled with Cy3 (Jackson) and anti-goat immunoglobulin labeled with FITC (Jackson). Cells were counterstained with DAPI. For imaging, Zeiss 63x oil immersion was used, and photographs were made of the human samples with a Zeiss Axioplan two microscope using Axiovision software. For the mouse samples, a Hamamatsu Orca AG microscope was used with Smart Capture Software.

### Two-electrode voltage clamp (*Xenopus* oocytes)

The cDNA sequence of a human *β*_2_-AR (Missouri S&T cDNA Resource Center) was subcloned from the pcDNA3.1 + to the pGEMHE *Xenopus* expression vector (kind gift of Prof. Jan Tytgat, Leuven). The pGEMHE vector was cut with BamHI (New England Biolabs) and HindIII (Roche). The *ADRB2* coding region was amplified with primers containing the aforementioned restriction sites, the amplicons were purified from a TAE-agarose gel, and cut with the same enzymes. The resulting product was ligated using the Ligafast™ Rapid DNA Ligation System (Promega) and the constructs were verified by sequencing (VIB Genetic Service Facility, University of Antwerp). They were cut with NheI (Roche) for linearization. The gene encoding the FLAG-tagged Cystic Fibrosis Transmembrane conductance Regulator (CFTR) ion channel was derived from the M2 901/pBQ4.7 vector (kind gift of Prof. Jan Eggermont, Leuven) and was linearized with XhoI (New England Biolabs) for *in vitro* transcription. RNA was then transcribed *in vitro* with the Ribomax kit (Promega) or the mMESSAGE mMACHINE T7 kit (Ambion) following the manufacturer’s protocol. RNA was visualized and its quality assessed on RNase-free agarose gels, and stored at −80°C. Buffers used for incubation of the oocytes were Ringer’s buffer (ORi): 90 mM NaCl, 3 mM KCl, 2 mM CaCl_2_, 5 mM HEPES, adjusted to pH 7.6 with NaOH, and a high potassium buffer: 5 mM NaCl, 65 mM KCl, 2 mM CaCl_2_, 5 mM HEPES and an equivalent amount of sugar or N-Methyl-D-glucamine (NMDG), ensuring equi-osmolarity between sugar-containing and sugar-free buffers. The maximum measured difference in osmolarity between the buffers was 3.6%.

Oocytes were collected and incubated as previously described (Weber et al., 1989; Weber et al., 1995). Briefly, for oocyte collection, female *Xenopus laevis* frogs from the stock of the laboratory of Prof. W. Van Driessche (Laboratory of Physiology, Medical faculty, KU Leuven) and purchased from African Xenopus Facility (Knysna, South Africa), were hypothermally anesthetized by submersion in ice water/crushed ice for 30 min. After abdominal incision, the ovarian lobes were pulled out, cut off, and the oocyte clump was placed in ORi. Oocytes were then liberated in ORi-collagenase (1 mg/ml) for 1–2 h on a shaking incubator, and subsequently transferred to a Ca^2+^-free solution using one washing step. They were shaken vigorously for maximum 15 min before collecting the individual oocytes in ORi buffer. Stage V oocytes were then selected under the microscope.

Voltage clamp measurements were performed as previously described (Amasheh and Weber, 1999; Weber et al., 1999; Weber et al., 2001a; Weber et al., 2001b). Briefly, for injection of the oocytes*,* RNA encoding the CFTR and/or *β*_2_-AR protein was drawn from a droplet afloat in mineral oil with a glass capillary. Injection volume was 46 nl. The amount of RNA varied between 10 and 25 ng. Injected oocytes were stored in ORi at 16°C for maximum 3 days until ready for testing. During the measurements, oocytes were continuously clamped to −60 mV. All measurements were recorded using the home-made software, DSPOOC.

### Assessment of *β*_2_-AR responses in cultured mammalian cells

A cDNA for the human *ADRB2* and *GNA15* was purchased from the Missouri S&T cDNA Resource Center and used for the construction of a stable Flp-In-293 (a derivative of the parent HEK293 cell line, Invitrogen) *β*_2_-AR/Gα16 cell line. This cell line was preferred over the STC1-cell line because of its low expression of *β*_2_-AR, so as to minimize possible interference with endogenous receptor signaling. The cell line expressing both *β*_2_-AR and Gα16 was selected in two successive transfection steps. First, Flp-In-293 cells were co-transfected with pMET7-ADRB2-FRT and pOG44. After Flp recombinase-assisted stable integration, an isogenic cell pool expressing the *β*_2_-AR was selected in hygromycin-containing medium (100 μg/ml). Second, the isogenic cell pool was cotransfected with p-GNA15 and pIRES-Puro2 (Clontech) in a 5:1 ratio, followed by selection of single clones in medium containing puromycin (.8 μg/ml). Flp-In-293 cells were maintained in DMEM medium (Gibco), containing penicillin/streptomycin (Sigma) and fetal bovine serum (Sigma) (10%). Hygromycin (100 μg/ml) was added for cells containing the *ADRB2* construct, and puromycin (1 μg/ml) was added for the cells containing the *GNA15* construct. The Flp-In-293 cells were transferred for *β*_2_-AR assays to clear- and flat-bottom 96-well plates in DMEM (Gibco) containing dialyzed fetal bovine serum (Sigma). The plates were coated with fibronectin (Sigma). For that purpose, the fibronectin solution was diluted 40 times in PBS, added in the plate wells (60 µl), and removed again after incubation for 1 h, after which the plates were allowed to dry for 1 h. The Fluo-4 NW kit (Invitrogen) was used for detection of calcium signals following the manufacturer’s protocol. All compounds tested were dissolved in HBSS (Sigma) at 37°C. Adrenergic antagonists (Sigma) were always added together with the Fluo4 compound, 50 min before addition of agonist. Agonists were added at time zero, 17 s after the start of the recording. Measurement of the calcium signals showed that they were generated about 5 s after addition of the agonists. The Flexstation II apparatus (Molecular Devices) was used to perform calcium measurements. After overnight growth of the cells in transfer medium in the aforesaid multiwell-96 plates, the medium was removed, the cells incubated with 100 µl of the loading dye solution (Fluo-4), the plates covered with aluminum foil, and incubated at 37°C for 30–50 min. Then, 50 µl of a 3x concentrated agonist solution (sugar or epinephrine) was added just prior to the measurement in the Flexstation II, to give a total volume of 150 µl per well. The data were normalized to the cell number in the individual wells.

### Response to epinephrine and mannose in Flp-In-293 cells stably transfected with *ADRB2* and *GNA15*, incubated in different HBSS-based buffers

This experiment was performed for checking the response to mannose in different conditions. Cells were incubated with Fluo-4 in different HBSS-based buffers either containing glucose 5 mM, mannose 5 mM or without glucose but supplemented with 4 mM L-glutamine (HBSS composition (mM): 1.26 CaCl_2_, .49 MgCl_2_, 5.33 KCl, .4 MgSO_4_, .44 KH_2_PO_4_, 137.9 NaCl, .34 Na_2_HPO_4_).

### Competitive radioligand binding assay

*β*_2_-AR-containing membranes (RBHBE2M) and ^125^I-cyanopindolol (NEX189) (spec. act. 2,200 Ci/mmol) were purchased from PerkinElmer. TRIS-HCl buffer containing MgCl_2_ and EGTA was used as incubation and assay buffer, while TRIS-HCl buffer was used as wash buffer. All buffers were cooled and all further steps performed on ice. Membranes were diluted 150 times in assay buffer. The radioligand was diluted to a final concentration of .097 nM and the tested compounds were added at the indicated concentrations. The mixtures were incubated for 1 h at ambient temperature, and subsequently filtered using a vacuum pump over GF/C filters (Whatman), pre-soaked in .5% polyethyleneimine, Following 9 rinses with ice-cold wash buffer, the filters were transferred to vials, which were read in a gamma-counter (Gamma master LKB Wallac 1277). Non-specific binding of ^125^I-cyanopindolol was determined with membranes devoid of *β*_2_-AR.

### Animals and diet

All the experiments were approved by the ethical committee on animal experimentation of the KU Leuven. Male or female Wistar rats (Harlan Netherlands B.V.) were maintained on a 12 h light-12 h dark cycle, and fed with standard rat food chow and water *ad libitum.*

### Preparation of everted intestinal sacs

Male or female rats weighing around 150 g (aged 6–7 weeks) were fasted overnight before the experiment, and euthanized by cervical dislocation on the day of the experiment. The abdomen was opened by a midline incision, and a segment of around 35 cm of proximal intestine was isolated. The intestinal segment was rinsed with ice-cold Ringer solution (composition (mM): 140 NaCl, 5 KCl, 1 MgCl_2_, 2 CaC1_2_, 10 HEPES, 10 TRIS, gassed with 95% O_2_ and 5% CO_2_, pH 7.4), and 4-7 everted sacs were prepared, each approximately 3 cm in length, by tying off the ends of the intestinal segments with threads (Hamilton and Butt, 2013). The sacs were filled with Ringer solution containing 5 mM mannitol (Sigma Aldrich) (to maintain osmotic balance) and L-glutamine (2 mM) (Sigma Aldrich) (as an energy source). Each everted sac was transferred to a separate glass beaker containing 50 ml continuously oxygenated Ringer solution with L-glutamine (2 mM), and maintained in a water bath at 37°C. Then, epinephrine 10 µM (Sigma Aldrich), mannose 5 mM (Sigma Aldrich), ICI 118,551 10 µM (Sigma Aldrich), phlorizin 100 µM (Sigma Aldrich), LX4211 2 µM (MedKoo Biosciences, Inc. United States) or Ringer solution were added to the respective beaker for a 15-min pre-incubation (concentrations indicate final concentrations of the compounds). For the sacs treated with ICI 118,551, phlorizin or LX 4211, the inhibitors were added before mannose or epinephrine. After pre-incubation, 5 mM glucose, or 2.5 mM glucose for the ICI 118,551 control experiment, was added to the external buffer to initiate glucose transport. After 10 min incubation, a sample was collected from inside the sac using a 1 ml syringe (Terumo) with a 26 gauge needle (Terumo). These samples were analyzed using a glucose assay kit based on glucose oxidase (Sigma Aldrich, for details, see below). Sacs treated with colchicine 5 µM (SERVA Feinbiochemica) or myristoylated PKI 14–22 amide 1 µM (Tocris), were pre-incubated for an additional 10 min before adding epinephrine or ICI 118,551. Also for colchicine, a TRIS-free Ringer solution was used, as TRIS interferes with colchicine activity (Margulis et al., 1969). For comparing glucose transport rates, each everted sac was first pre-incubated for 15 min in Ringer solution, then glucose was added to a final concentration of 10 mM for a further incubation during 5, 10 or 75 min. We noticed that it was important to take certain precautions during these experiments: the intestine was never allowed to overfill and be stretched while rinsing the intestinal lumen at the time of isolation and filling the everted sac with Ringer solution. Trapping air bubbles inside the everted sac was avoided. Also, the regions of the intestine where mucus was still present were not used for preparing everted sacs.

### Glucose assay

The glucose concentration of the samples was assayed by a glucose assay kit (Sigma Aldrich, GOD/POD method). The assay procedure recommended by the kit was modified for small sample volumes as follows. Fifty µL of sample was transferred to a well of a 96-well plate, and at time zero, the reaction was started by adding 100 µl of assay reagent to the first well, and mixing. Each well was allowed to react for exactly 30 min at 37°C. The reaction was stopped by adding 100 µl of 12 N H_2_SO_4_ (Merck) into each well, and carefully mixing. The absorbance was measured at 540 nm with a plate reader (Tecan 200). Mannose is only detected by the glucose oxidase assay with about 100-fold lower sensitivity than for glucose (Raba and Mottola, 1995).

### *In vivo* oral glucose tolerance test

The oral glucose tolerance test was performed on normal male or female rats weighing around 250 g (aged 8–11 weeks), which were fasted for 16 h before the test. Blood was sampled by tail vein puncture, and the blood glucose level was measured by glucometer (Verio OneTouch glucometer). Rats were divided in four groups: one with glucose 2 g/kg or glucose 4 g/kg (Sigma Aldrich), both in the presence or absence of CD3-403. The CD3-403 compound displays very low cell permeability (assessed by a Caco-2 permeability assay, as described in more detail in Supporting Information Table S2), and hence we aimed for a local intestinal concentration of 5 μM, based on 10 times the IC_50_ and using an additional safety factor of 10 for any non-specific binding to intestinal content such as mucus. Glucose or glucose along with CD3-403, dissolved in .9% saline, was administered to rats by oral gavage in the respective groups. Blood glucose was measured at 0, 15, 30, 60, and 120 min. All experiments were conducted around the same time in the morning.

### STD-NMR

Cell membranes were prepared as described in Hoare *et al.*(Hoare and Usdin, 1999), with some modifications. Flp-In-293 cells (derived from the parent HEK293 cell line) stably transfected with *ADRB2* and *GNA15* expression constructs were grown in DMEM medium, containing penicillin (100 U/ml)-streptomycin (100 μg/ml) and fetal bovine serum (10%). Hygromycin (100 μg/ml) was added for the *ADRB2* construct and puromycin (1 μg/ml) was added for the *GNA15* construct as mentioned in the methods section for the calcium assay. Non-transfected HEK Flp-In 293 cells, which have a low endogenous expression of the *β*_2_-AR, were grown in culture medium as described above, but without hygromycin and puromycin. After reaching confluence in a culture flask of 150 cm^2^, monolayers of both cell lines were dislodged by trituration with their respective culture media. Cells were centrifuged at 150 g, 20°C for 4 min, the supernatant medium was discarded, and the cells were washed with PBS. The cells were then re-suspended in 40 ml lysis buffer (25 mM TRIS, 2 mM EDTA, 6 mM MgCl_2_ and .1 mM 4-(2-aminoethyl) benzenesulfonyl fluoride hydrochloride (AEBSF) pH 7.5, for 4 confluent flasks), kept at 4°C, and homogenized in an ice-cold glass Dounce homogenizer with 45 strokes. The homogenates were centrifuged at 1,000 g, 4 °C for 10 min to remove intact cells. The supernatants were centrifuged at 40,000 g, 4°C for 30 min. To ensure that the cell membrane preparations were guanine-nucleotide-free, the resulting pellet was washed with 30 ml lysis buffer, and finally suspended in buffer with 20 mM HEPES, 100 mM NaCl, 1 mM EDTA and 3 mM MgSO_4_, pH 7.5. Total protein was quantified with the bicinchoninic acid assay (BCA assay) using bovine serum albumin (BSA) as the standard. Cell membranes were stored at −80°C until use. Membranes were reconstituted in PBS for the STD-NMR assay.

NMR experiments were performed at 5°C on a Bruker Avance II 600 NMR spectrometer equipped with a cryogenic TCI probe with a z-gradient. The standard Bruker pulse program stddiffesgp (Mayer and Meyer, 1999) was used for data collection using excitation sculpting to suppress the water signal and a 5-s STD saturation time. Data are collected with 32 k complex points for 2.5 s. A delay of 2 s is applied between each FID to ensure complete relaxation. For each of the STD experiments, 32 scans are accumulated. The spectra for both on-resonance and off-resonance saturation at resp. .7 and 12 ppm are collected interleaved. The Bruker command stdsplit was used to process and subtract on-and off-resonance FIDs.

### Statistical analyses

For the experiments on epinephrine and mannose stimulation of glucose transport and their inhibition by ICI 118,551, and for the experiments on the effect of LX 4211 and phlorizin on glucose and mannose transport, a one-way ANOVA was performed, followed by Tukey’s test. For the experiment on the effect of colchicine on epinephrine stimulation of glucose transport, the direction of the effect of colchicine (inhibition) and epinephrine (stimulation) was known, hence, a one tailed *t*-test was applied. For the effect of ICI 118,551 on glucose transport, a two-tailed *t*-test was applied. In case of the *in vivo* glucose bolus administration, the direction of the effect that we wanted to test was known, i.e. inhibition by CD3-403, hence a one tailed *t*-test was applied. All the statistical analyses were performed using GraphPad Prism 5 software.

## Results

### The *β*_2_-AR is expressed at the apical membrane of enterocytes

We have first tested which GPCRs are expressed in enterocytes (Table 1), in order to investigate subsequently those located in the apical membrane for a possible sugar-sensing function. We first used PCR amplification of selected GPCRs (Vassilatis et al., 2003) starting from cDNA derived from mouse proximal duodenal mucosal scrapings, and from cells of the intestinal STC-1 cell line (Secretin Tumor Cell line - 1) incubated in 5 mM or 25 mM glucose. Twenty-five GPCRs could be amplified in this way from the two sources together (Table 1). It has to be mentioned that mucosal scrapings are likely not only composed of epithelia but might also contain some submucosal tissue. Hence, some of the GPCRs detected with the mucosal scrapings might be derived from submucosal tissue. We then performed microarray gene expression analysis and SYBR Green qPCR of STC-1 cells incubated in 5 mM or 25 mM glucose. We identified 22 GPCRs with the microarray gene expression analysis and using the SYBR Green qPCR approach we also detected some additional GPCRs that were not present on the microarray (Table 1). Finally, using RNA from STC-1 cells we confirmed by qPCR the expression of 16 GPCRs detected by the microarray gene expression analysis (Table 1).

**TABLE 1:** GPCRs whose expression was detected in mouse proximal duodenal mucosal scrapings or in the intestinal STC-1 cell line cultured in medium with 5 or 25 mM glucose for the last 24 h.

	PCR			Microarray		qPCR	
	Mouse mucosal scrapings	STC-1 cell line		STC-1 cell line		STC-1 cell line	
		5 mM Glu	25 mM Glu	5 mM Glu	25 mM Glu	5 mM Glu	25 mM Glu
5-HT1b				+	+		
5-HT3a				+	+		
ADRB2	+	+		/	/		
AGTRL1	+		+	−	−	+	+
ATIIR				−	+		
celsr3				+	+		
GPR1	+	−		+	+		+
GPR120	+	−		+	+		+
GPR125	+	+		/	/		+
GPR151	+		+	/	/	+	+
GPR18	−	−		+	+		+
GPR19	+	+		+	+		+
GPR22	−	−		/	/		+
GPR35	+		+	/	/	+	+
GPR39	+	−		+	+		+
GPR40	+	+		/	/		+
GPR43	+	+		+	+		
GPR48	+	+		+	+		+
GPR49	−	+		+	+		+
GPR55	−		−	/	/		
GPR56	+	+		+	+		+
GPR61	+	−		+	+		+
GPR73	−		−	−	−		
GPR80	+	−		/	/		+
GPR85	+	+		+	+		+
GPR88	−	−		+	+		+
GPRC5C	+		+	+	+	+	+
PGR22	+		+	/	/		
prosta E4				+	+		
RDC1	+		+	−	−	+	+
TEM5	−		+	−	−	+	+
TM7SF1	+	−		+	+		+
TM7SF2	+	+		+	+		+
TM7SF3	+	+		+	+		+
TPRA40	+		+	+	+	+	+

+, positive detection; −, no detection;/, not present on microarray; blank table cells: not tested; Glu, glucose.

**FIGURE 1:**
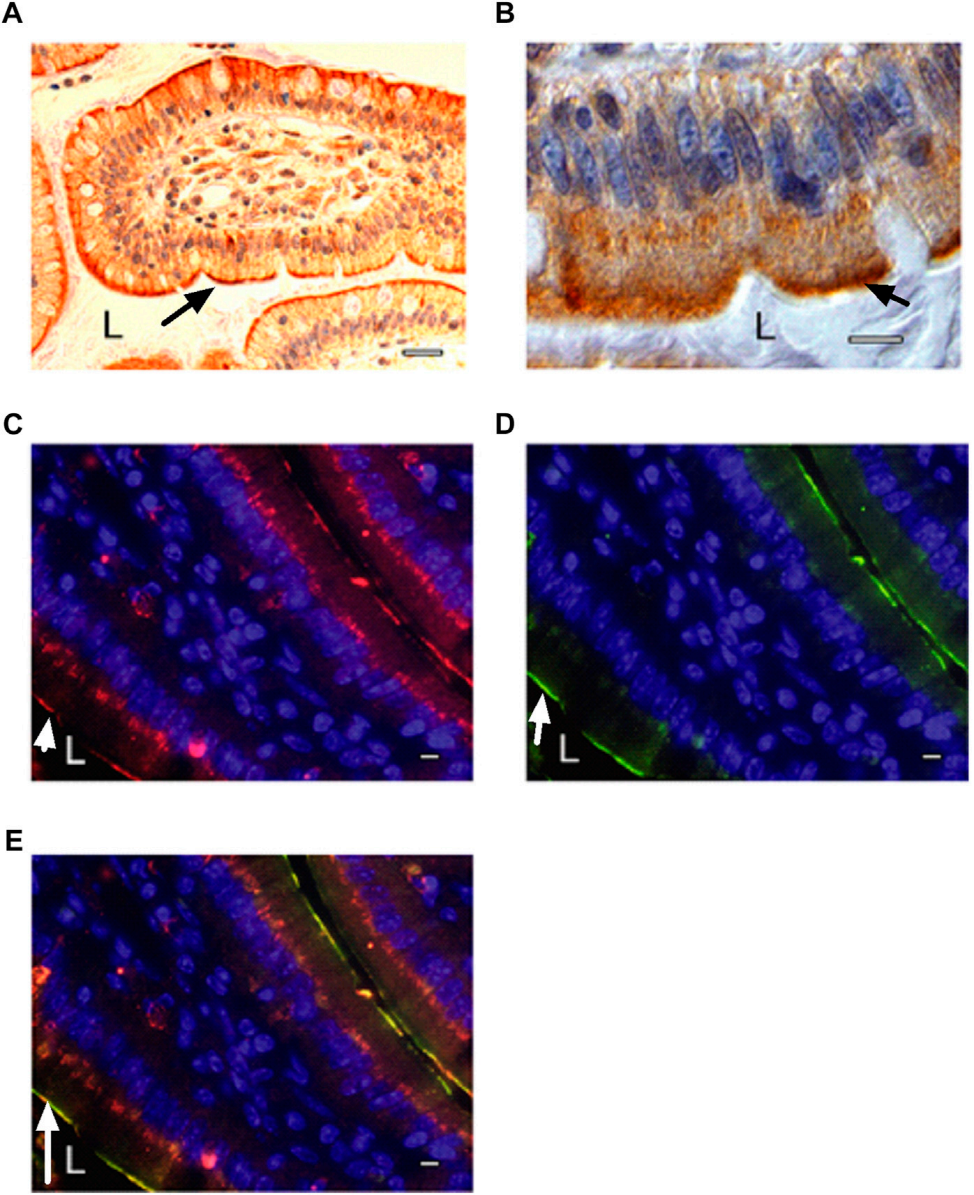
Localization of *β*_2_-ARs at the apical side of intestinal epithelial cells. **(A)** *β*_2_-AR immunostaining using Abcam antibodies in human duodenal biopsy sample as revealed by peroxidase staining (L: lumen of intestine). Secondary antibodies were peroxidase-labeled goat anti-rabbit antibodies (Abcam). The terminal part of a villus is shown with some surface sections of adjacent villi. **(B)** magnification of *β*_2_-AR immunostaining in individual epithelial cells (L: lumen of intestine). The strong staining on the apical plasma membrane is clearly visible as well as the more diffuse staining inside the enterocytes, possibly in ER/Golgi. The nucleus is colored blue with DAPI. **(C–E)** immunostaining in mouse intestinal tissue sections of **(C)** *β*_2_-AR with rabbit antibodies (Assay Design) and CY3-conjugated secondary anti-rabbit antibodies (Jackson) (red) and **(D)** SGLT1 with goat antibodies (M19, Santa Cruz) and FITC-conjugated secondary anti-goat antibodies (Jackson) (green), **(E)** overlay showing co-localization of *β*_2_-AR and SGLT1. The nucleus is colored blue with DAPI. A single villus is seen in the middle of the panels, with one side adjacent to lumen at the bottom left and showing a single line of *β*_2_-AR or SGLT1 apical staining on the enterocytes of that side. On the other side of the villus, at the upper right corner, it is just adjacent of a next villus with very little lumen in between the two villi, so that a double line of two apical plasma membranes with staining of *β*_2_-AR or SGLT1 is visible. SGLT1 staining is largely confined to the apical plasma membrane while *β*_2_-AR staining is also present in more diffuse form at the bottom of the epithelial cells, possibly in the ER/Golgi. The arrow indicates either *β*_2_-AR **(A–C)**, SGLT1 **(D)** or co-localization of the two **(E)**. The scale bar is 100 μm **(A)** or 10 μm **(B–E)**. All fluorescent images were taken under similar electronic amplification conditions. Representative results are shown from several samples investigated in all cases.

Next, we used immunohistochemistry on human biopsy samples from the duodenal part of the small intestine to determine if any of the GPCRs were located in the apical membrane. We first tested GPCRs for which antibodies were available. Among the first GPCRs tested: GPR105, GPR1, GPR120, GPRC5C and the *β*_2_-AR, only the last one surprisingly showed staining at the apical cell membrane (Supporting Information Figure S1A). The apical staining of the *β*_2_-AR was very strong and highly reproducible, compared to the much weaker staining with some of the other GPCRs (Figure 1A) (Supporting Information Figure S1A,B). These either showed staining at the basolateral membrane or showed staining too weak for reliable detection. Some much weaker cytoplasmic staining on the basal side of the enterocytes was also observed for the *β*_2_-AR, possibly in organelles of the secretory pathway (Figure 1B) (Supporting Information Figure S1B). Because the apical location of the *β*_2_-AR (facing the luminal side of the gut) is highly unusual for a receptor sensing a hormone distributed through the bloodstream and since the *β*_2_-AR is involved in glucose and energy homeostasis, and couples to the cAMP-PKA signaling pathway, we further concentrated on the possibility that the *β*_2_-AR might have an additional glucose-sensing function. The apical localization of *β*_2_-ARs in enterocytes was confirmed with paraffin-embedded tissue sections of proximal mouse intestine (Figure 1C), and showed near complete overlap with immunoreactivity for SGLT1, which is specifically located in the apical membrane (Figures 1D,E). This, however, does not necessarily indicate that the two proteins would physically interact. Human and mouse *β*_2_-AR show 87% sequence identity (https://www.uniprot.org/align/A20200503DA437993067D6F64326E5E763500BDED0207523). Our immunohistochemistry results demonstrating the apical localization of the *β*_2_-AR in intestinal epithelial cells are consistent with previous results in the literature. Singh et al. (2009) reported the mRNA expression level for *β*_2_-AR in murine duodenal epithelial cells, as well as the strong enrichment of *β*_2_-ARs by Western blotting in the apical brush border membrane compared to the total cell lysate. Both *β*_2_-AR bands (monomer and dimer) were completely blocked in the Western blot using the immunising peptide, showing the specificity of their antibody (Singh et al., 2009).

### The *β*_2_-AR responds to sugars and this response is blocked by *β*-AR antagonists

**FIGURE 2:**
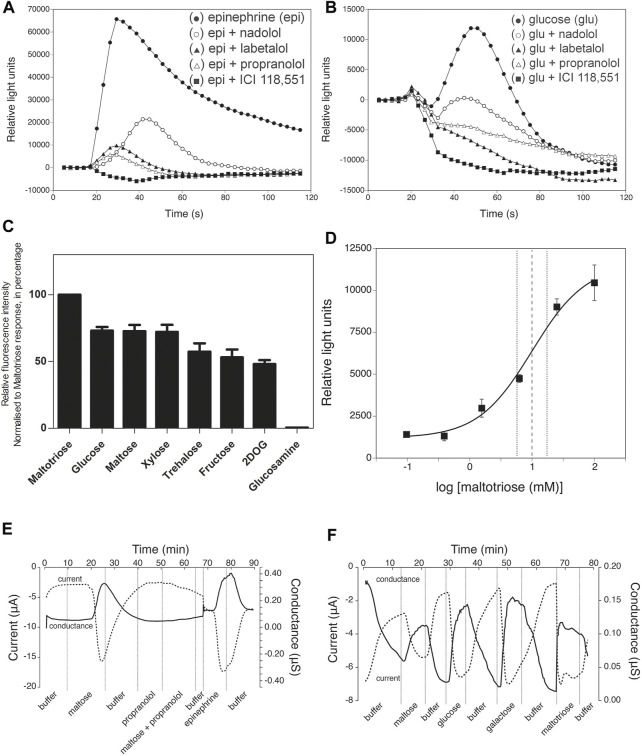
Inhibition by *β*-AR antagonists, sugar specificity and affinity of *β*_2_-AR. **(A)** Epinephrine- (20 nM, black filled circle) and **(B)** glucose- (70 mM, black filled circle) induced responses and their inhibition by pre-incubation for 50 min together with the Fluo4 compound with various *β*-AR antagonists (all at 200 μM): nadolol (*β*_1,2_-antagonist, white open circle), labetalol (*α,β*-antagonist, black filled trangle), propranolol (*β*_*1,2*_-antagonist, white open triangle) and ICI 118,551 (*β*_2_-antagonist, black filled square) of *β*_2_-AR expressed in Flp-In-293 cells. Addition of agonists was done at time zero, 17 s after the start of the recording of the calcium signals generated. Note the difference in *Y*-axis scale between **(A)** and **(B)**. Representative results from at least three repetitions are shown. **(C)** Specificity of the response to different sugars added at 50 mM. In all cases, except for 2-deoxyglucose (2DOG), the responses were completely blocked by 1 µM ICI 118,551. For 2DOG, only the response blocked by ICI 118,551 is shown. The *y*-axis displays peak fluorescence intensity (arbitrary units) for the response to the different sugars. All values are expressed as mean ± SD (*n* = 3; technical replicates). **(D)** Dose-response curve for maltotriose activation of the *β*_2_-AR expressed as Relative Light Units as a function of the log maltotriose concentration. The EC_50_ is approximately 10.00 mM. Maltotriose was used instead of glucose because of rapid uptake of glucose in the cells, while maltotriose is not taken up by the HEK Flp-In 293 cells. The derived curve is the result of 3 independent, biological repeats. The dashed vertical lines indicates 95% confidence interval on the calculated EC_50_. All values are expressed as mean ± SD. **(E)** Maltose and epinephrine responses of *β*_2_-AR expressed in *Xenopus* oocytes. Propranolol does not trigger a response itself, but prevents the maltose-induced response. **(F)** Responses to different sugars of *β*_2_-AR expressed in *Xenopus* oocytes. (**E,F)** for composition of the buffer: see Materials and Methods).

We subsequently constructed a stably transfected Flp-In-293 kidney cell line overexpressing a human *β*_2_-AR, in which receptor activation is coupled *via* Gα16 to phospholipase-C-mediated Ca^2+^ release from intracellular stores, as detected by Fluo4 fluorescence. These cells responded as expected to catecholamine agonists, like epinephrine (20 nM), and the response was blocked by classical *β*-AR antagonists (Smith and Teitler, 1999; Baker, 2005), like nadolol (β1,2-antagonist), labetalol (α,β-antagonist), propranolol (β1,2-antagonist) and ICI 118,551 (β2-antagonist) (Figure 2A). Control experiments with HEK Flp-In 293 cells without transfection of the *β*_2_-AR showed only a minimal background calcium response with epinephrine, indicating that any endogenous *β*_2_-AR expression was too low to give a significant response with our reporter system (Supporting Information Figure S2). Addition of ICI 118,551 alone did not trigger a significant change in the fluorescence read-out, with epinephrine (5 nM) and Hank’s Buffered Salt Solution (HBSS) used as positive and negative control, respectively (Supporting Information Figure S3). Using this reporter system, we next evaluated whether sugars were able to trigger activation of the *β*_2_-AR. Glucose (70 mM) triggered a rapid, but less pronounced response compared to epinephrine, that was completely inhibited by the aforementioned *β*-AR antagonists (Figure 2B). ICI 118,551 was equally effective in blocking the epinephrine and glucose responses at 30 μM. Other sugars also activated the *β*_2_-AR in order of decreasing intensity: maltotriose, glucose, maltose, xylose, trehalose, fructose and 2 deoxy-D-glucose. all used at 50 mM (Figure 2C), suggesting different degrees of agonist potency. Glucosamine (50 mM) provoked very little effect. The time-dependency of the different sugar responses was very similar and ICI 118,551 inhibited all sugar responses at 1 μM (see further in the description of Figure 4). The sugar concentrations are in the same range as their estimated concentrations after a meal in the gut of mammals, including humans (Olsen and Ingelfinger, 1968; Ferraris et al., 1990). Rapid cell uptake of glucose at low concentrations hampered accurate determination of the EC_50_ of the *β*_2_-AR for glucose. For maltotriose, a sugar not taken up by the cells, an EC_50_ of approximately 10 mM was determined (Figure 2D). (The calculated EC_50_ was 10.05 mM, with a 95% confidence interval between 5.06 mM and 19.93 mM. Quality of the curve fitting was characterized by an R squared of .93).

To exclude that the genetically engineered Gα16/phospholipase-C mediated Ca^2+^ signaling was in some way affecting the specificity of the *β*_2_-AR, we also used a cAMP-dependent reporter system. For that purpose, we expressed *β*_2_-ARs in *Xenopus* oocytes, as has previously been reported (Kobilka et al., 1987), but now together with a reporter system consisting of the PKA-dependent cystic fibrosis transmembrane conductance regulator (CFTR) Cl^−^channel. Thus, parallel changes in inward transmembrane current and conductance of the membrane served as readout for receptor activity. Here as well, both epinephrine and different sugars activated the *β*_2_-AR (Figures 2E,F) while the *β*-AR antagonist propranolol inhibited the responses (Figure 2E). There was neither an epinephrine nor a sugar response in control *Xenopus* oocytes injected with vector mRNA, while addition of forskolin (activator of adenylate cyclase) together with IBMX (inhibitor of cAMP phosphodiesterase) produced a strong response (Supporting Information Figure S4).

### Sugars do not compete with antagonist binding to *β*_2_-AR

**FIGURE 3:**
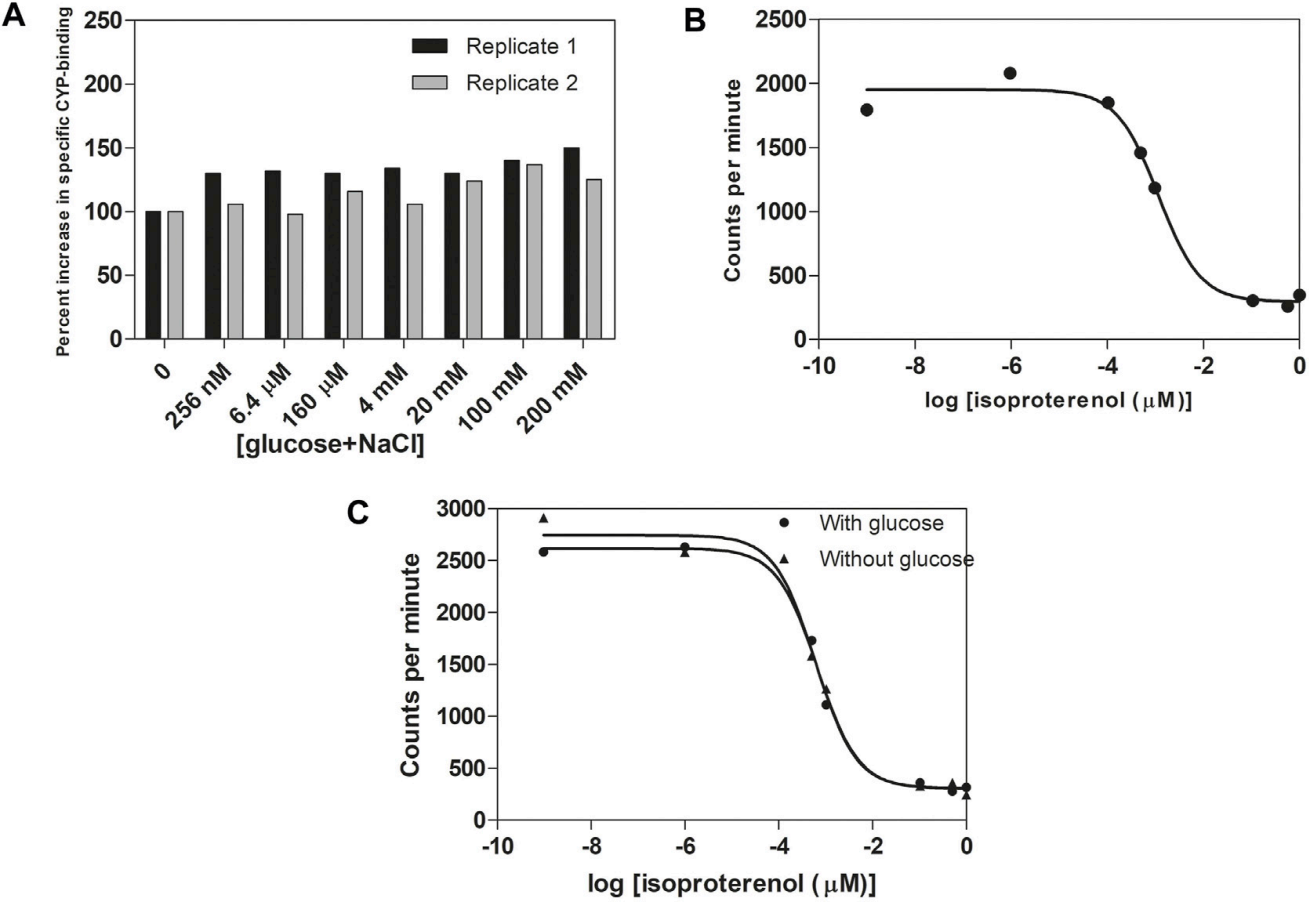
Glucose, as opposed to isoproterenol, does not compete with ^125^I-cyanopindolol binding to cell-free isolated membrane vesicles containing *β*_2_-AR. **(A)** Stimulation of ^125^I-cyanopindolol binding with increasing equimolar concentrations of glucose + NaCl (with the total concentration indicated on the *X*-axis). Two independent replicates were used from separate experiments. Binding in the absence of glucose (2000–2,700 cpm) was normalized to 100%. Equal volumes of membrane suspension were used. **(B)** Inhibition of ^125^I-cyanopindolol binding by increasing concentrations of the *β*-AR agonist, isoproterenol. The log values at -9 and -6 represent basal binding of ^125^I-cyanopindolol. **(C)** Indirect competition assay between glucose and isoproterenol for binding of ^125^I-cyanopindolol to *β*_2_-AR. Addition of 100 mM glucose had no significant effect on the inhibition by any concentration of isoproterenol for ^125^I-cyanopindolol binding to *β*_2_-AR.

We next investigated whether the two types of agonists, epinephrine and sugars, bind to the same ligand-binding site. For that purpose, we determined binding of ^125^I-cyanopindolol, a *β*-AR antagonist known to bind into the epinephrine binding site of the β2-AR (Cook et al., 1985; Kobilka et al., 1988; Warne et al., 2008; Chan et al., 2016; Masureel et al., 2018; Warne et al., 2019), to isolated cell-free membrane vesicles containing recombinant human *β*_2_-AR (Perkin Elmer Cat. nr. RBHBE2M) in the absence or presence of glucose. The two ligands were used at physiologically relevant concentrations. The large difference in affinity for epinephrine/cyanopindolol compared to glucose fits with the large difference in the concentration of epinephrine in the blood and that of glucose in the gut lumen, as well as in the blood, to which the *β*_2_-AR should be able to respond appropriately. We actually tested the effect of glucose alone, glucose + NaCl and NaCl alone, because of a previous report that NaCl by itself enhanced ^125^I-cyanopindolol binding (Minuth and Jakobs, 1986). We did not observe any inhibition of ^125^I-cyanopindolol binding in the presence of glucose + NaCl (Figure 3A) or glucose alone (Supporting Information Figure S5A), suggesting that cyanopindolol and glucose do not bind in the same site on the *β*_2_-AR, in spite of our previous observation that the glucose response was blocked by a range of classical *β*-AR antagonists (Figure 2B). Surprisingly, we even observed an increase in ^125^I-cyanopindolol binding with up to about 40% in the presence of glucose + NaCl (Figure 3A) and up to about 30% in the presence of glucose alone (Supporting Information Figure S5A). This suggests that glucose binding affects the structure of the epinephrine-binding site. The affinity of this glucose response, however, should not be confused with the affinity by which *β*_2_-AR triggers activation of the G_s_-protein upon binding of glucose alone. In that case indeed there is no other compound bound into the epinephrine binding site. The stimulation of ^125^I-cyanopindolol binding by glucose merely confirms that glucose physically interacts with *β*_2_-AR although it also suggests that glucose could modulate epinephrine binding *in vivo*. In agreement with the previous report (Minuth and Jakobs, 1986), we also observed stimulation of ^125^I-cyanopindolol binding with increases up to roughly 35% in the presence of increasing concentrations of NaCl (Supporting Information Figure S5A). In a control experiment, increasing concentrations of isoproterenol, a structural analog of epinephrine and non-selective *β*-AR agonist, caused the expected gradual inhibition of ^125^I-cyanopindolol binding (Figure 3B). Addition of glucose also had no significant effect on the inhibition by isoproterenol of ^125^I-cyanopindolol binding (Figure 3C). These results appear to indicate that sugars and epinephrine may not bind into precisely the same site, although binding of the sugar does appear to affect the structure of the epinephrine binding site. This agrees with the demonstration of conformational coupling between the epinephrine binding site and an allosteric binding site for other ligands on the extracellular surface of the *β*_2_-AR (Bokoch et al., 2010). Because both the epinephrine and sugar responses of the *β*_2_-AR are inhibited by classical *β*-AR antagonists (Figures 2A,B), the two binding sites may be located close to each other or may affect each other over a greater distance by the reported conformational coupling upon ligand binding. In recent years, an increasing number of allosteric ligands has been discovered for GPCRs. They bind to allosteric sites, as opposed to the orthosteric ligands that bind to the binding site for the native ligand (Canals et al., 2011). On the other hand, it is premature at present to suggest that glucose and adrenergic agonists would bind to two entirely different sites, or that glucose would bind to an allosteric site rather than the orthosteric site, especially because of the consistent inhibition of all sugar responses by *β*-AR antagonists.

### Some *β*-AR antagonists differentially inhibit *β*_2_-AR responses to epinephrine, glucose and other sugars

**FIGURE 4:**
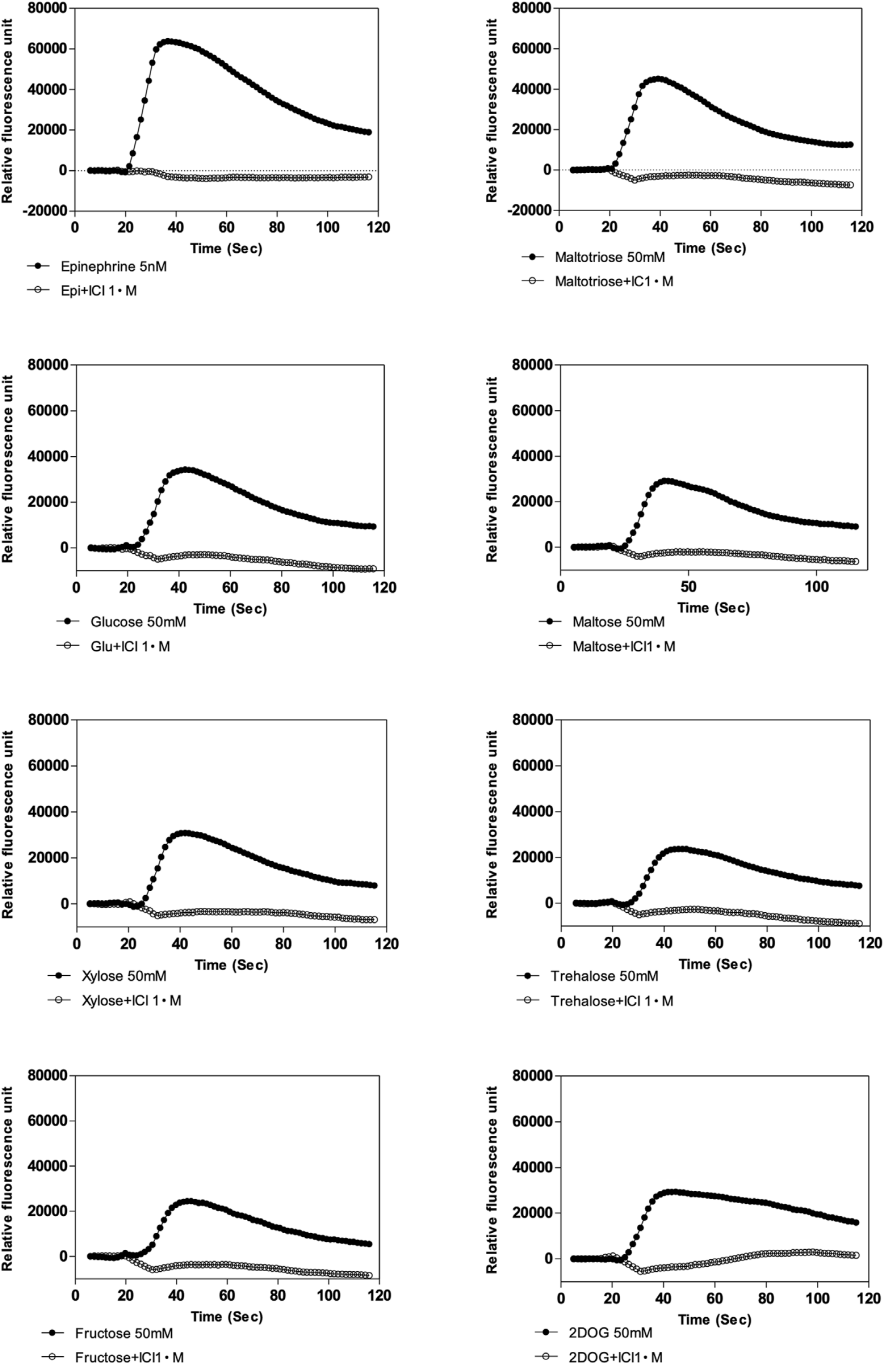
Effect of *β*_2_-specific antagonist, ICI 118,551 on calcium response elicited by epinephrine (5 nM) and various sugars (50 mM) in Flp-In-293 cells stably transfected with ADRB2 and GNA15. One µM ICI 118,551 inhibits epinephrine- and sugar-elicited *β*_2_-AR -mediated calcium responses. *X*-axis shows time in s, and *y*-axis displays fluorescence intensity (arbitrary units). Representative results are shown from at least three repetitions.

Because the two types of ligands, epinephrine and sugar, may bind to sites on the *β*_2_-AR that differ at least to some extent, we tested several *β*-AR antagonists in search of compounds with a possible differential effect on the two ligand-binding sites. The responses to all sugars tested, i.e. maltotriose, glucose, maltose, xylose, trehalose, fructose and 2-deoxy-D-glucose, which showed a very similar time-dependency, were all inhibited by β2-antagonists: 1 μM ICI 118,551 (Figure 4), propranolol, labetalol and nadolol (Supporting Information Figure S6). In contrast, β1-specific antagonists, even at a concentration of 200 μM, did not inhibit the epinephrine response (Smith and Teitler, 1999; Baker, 2005) (Supporting Information Figure S7) but differentially affected the response to sugars. Metoprolol (Supporting Information Figure S7), acebutolol or atenolol (Supporting Information Figure S8) at 200 μM completely inhibited the response to 70 mM glucose, but only slightly affected the response to 70 mM maltose, 2-deoxy-glucose or xylose (Supporting Information Figures S7, S8). Also the results in Supporting Information Figure S6 show that the glucose response is more sensitive to the different β2-antagonists than the response to the other sugars, which even at a concentration of 200 μM is often not completely blocked. These results are consistent with binding of epinephrine and glucose to two different sites. The stronger antagonism of β1-antagonists for the glucose response compared to the other sugars, at least under the conditions of our experiments, suggests that glucose binds somewhat differently to the *β*_2_-AR than the other sugars. It may suggest that glucose is the natural endogenous ligand of the *β*_2_-AR for sugar activation, and that the other sugars only activate fortuitously because of structural similarity.

### Everted sacs of rat intestine show sugar-induced, *β*_2_-AR-dependent stimulation of glucose uptake

**FIGURE 5:**
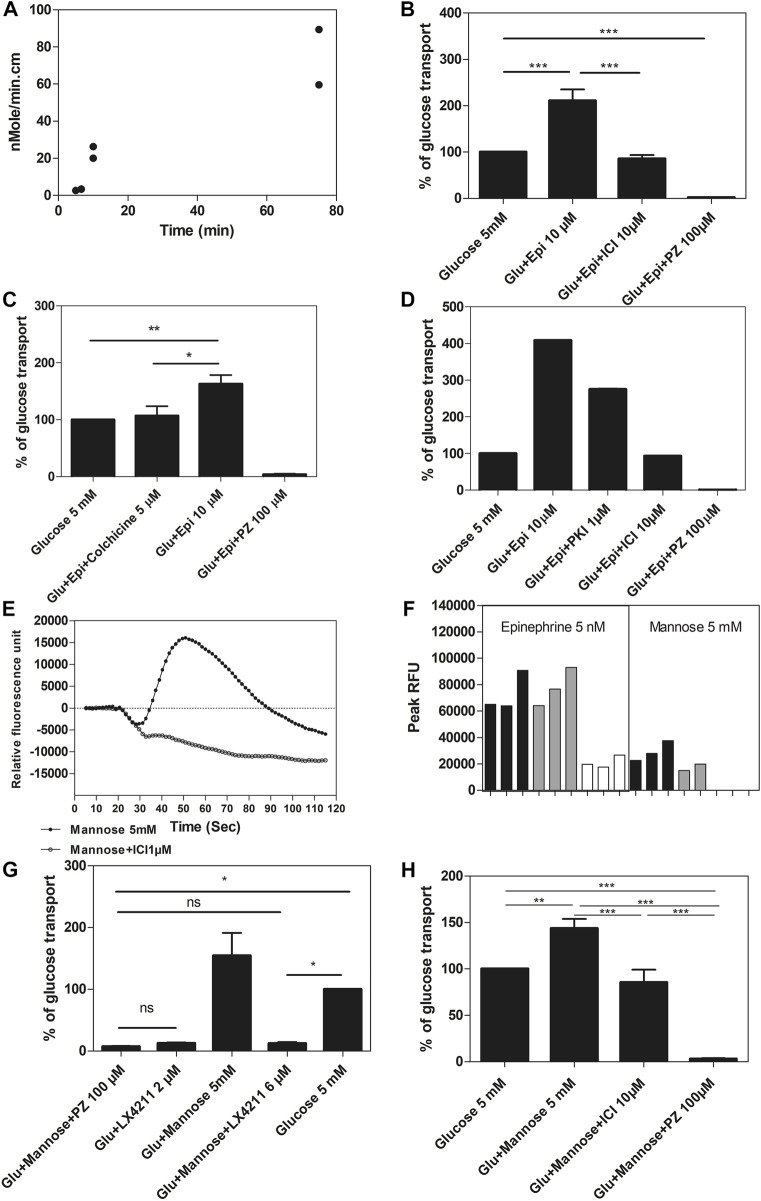
Glucose transport in everted sacs prepared from rat intestine. **(A)** Comparison of the amount of glucose accumulated per min in the everted sacs, prepared from rat intestine (*n* = 2; biological replicates), during different time periods (5, 10, and 75 min) after addition of 10 mM glucose. The data represented as individual replicates. **(B)** Effect of epinephrine (Epi), the β2-specific antagonist ICI 118,551 (ICI) and the glucose transport inhibitor phlorizin (PZ) on the level of glucose (Glu) accumulated after 10 min inside everted sacs, prepared from rat intestine. ICI 118,551 blocks epinephrine-stimulated glucose transport. (Respective number of replicates *n* = 16, *n* = 10, *n* = 7, *n* = 6 for conditions shown in figure). **(C)** Effect of epinephrine (Epi), colchicine and phlorizin (PZ) on the level of glucose (Glu) accumulated after 10 min inside everted sacs prepared from rat intestine: colchicine inhibits Epi-stimulated Glu transport. (Respective number of replicates *n* = 3, 5, 3, and 3 for conditions shown in figure). **(D)** Effect of epinephrine (Epi), the PKA inhibitor myristoylated PKI 14–22 amide (PKI), ICI 118,551 (ICI) and phlorizin (PZ) on the level of glucose (Glu) accumulated after 10 min inside everted sacs prepared from rat intestine: myristoylated PKI 14–22 amide inhibits Epi-stimulated Glu transport partially, while ICI 118,551 inhibits completely. (Respective number of replicates *n* = 2, 2, 4, and 2 for conditions shown in figure). **(E)** Calcium response elicited by 5 mM mannose in Flp-In-293 cells, stably transfected with *ADRB2* and *GNA15*, and incubated in Hank’s Balanced Salt Solution (HBSS) throughout with glucose 5 mM (representative graph from *n* = 3). Mannose elicits a *β*_2_-AR-mediated calcium response. *X*-axis shows time in seconds and *y*-axis displays fluorescence intensities (arbitrary units) for 5 mM mannose and 5 mM mannose +1 µM ICI 118,551. **(F)** Calcium response elicited by 5 nM epinephrine or 5 mM mannose in Flp-In-293 cells, stably transfected with ADRB2 and GNA15, and incubated in different modifications of Hank’s Balanced Salt Solution (HBSS), either with 5 mM glucose (black bars) or 5 mM mannose (grey bars) throughout, or with no added sugar but 4 mM glutamine (as energy source) throughout (white bars). Mannose elicits a *β*_2_-AR-mediated calcium response in HBSS containing either glucose or mannose throughout but not when either sugar is replaced by glutamine. *X*-axis shows different conditions: addition of 5 nM epinephrine or 5 mM mannose in HBSS with (1) 5 mM glucose (black) or (2) mannose (grey) or (3) with sugar replaced by glutamine (white) (*n* = 2–3); *y*-axis represents peak relative fluorescence intensity (RFU, arbitrary units). **(G)** Effect of phlorizin (PZ) and the dual inhibitor of SGLT1 and SGLT2, LX4211, on the level of glucose (Glu) accumulated after 10 min inside everted sacs, prepared from rat intestine. The presence of mannose enhances the level of glucose transported into the everted sacs. Mannose is virtually not detected by the glucose oxidase assay for glucose (about 100-fold lower sensitivity). (Respective number of replicates *n* = 7, 3, 9, 11, and 6 for conditions shown in figure). **(H)** Effect of mannose, ICI 118,551 (ICI) and phlorizin (PZ) on glucose (Glu) accumulated after 10 min inside everted sacs, prepared from rat intestine. ICI 118,551 inhibits mannose-stimulated Glu transport. (Respective number of replicates *n* = 12, 12, 7, and 6 for conditions shown in figure.) Data information: The glucose transport for the different groups was expressed as a % of the glucose transport of the “glucose alone” group (for panel b, c, d, g and h). All values are expressed as mean ± SEM (or ±SD in panel a), *p* < .001:***; *p* < .01: **; *p* < .05: *; *p* > .05: ns. For the experiments on epinephrine and mannose stimulation of glucose transport and their inhibition by ICI 118,551, and for the experiments on the effect of LX 4211 and phlorizin on glucose and mannose transport, a one-way ANOVA was performed, followed by Tukey’s test. For the experiment on the effect of colchicine on epinephrine stimulation of glucose transport, the direction of the effect of colchicine (inhibition) and epinephrine (stimulation) was known, hence, a one tailed *t*-test was applied.

The importance of the apical *β*_2_-AR for sugar-induced stimulation of glucose transport in the gut was evaluated with an everted sac model, in which active glucose transport occurs from the external medium outside the sac, facing the intestinal mucosa, to the inside of the sac flanked by the serosa (Alam et al., 2012; Hamilton and Butt, 2013). Glucose from the external medium (5 mM) indeed accumulated inside the everted sac with a rate that strongly increased with longer incubation times in the presence of glucose (Figure 5A). Glucose uptake into the everted sacs was inhibited for more than 90% by phlorizin (100 μM) and by LX4211 (2 μM), inhibitors of both SGLT1 and SGLT2 (Supporting Information Figure S9). When epinephrine (10 µM) was added to the external medium, the amount of glucose accumulated after 10 min in the sac increased from 440 ± 132 to 833 ± 255 µM. This increase was completely prevented by the *β*_*2*_-AR antagonist ICI 118,551 (10 μM), while phlorizin (100 μM) inhibited both basal and enhanced glucose uptake (Figure 5B). This supports a role of the apical *β*_2_-AR in stimulating glucose uptake through SGLT from the gut. Colchicine (5 μM), a microtubule-disrupting agent that blocks translocation of SGLT1 from a cytoplasmic pool to the apical plasma membrane (Yu et al., 2008), completely blocked epinephrine stimulation of glucose transport, but not basal glucose uptake, as was observed with phlorizin (100 μM) (Figure 5C). This suggests that epinephrine acts by increasing translocation of SGLT1 from a cytoplasmic pool to the enterocyte apical surface. Myristoylated PKI 14–22 amide (mPKI) (1 μM), a selective cell-permeable protein kinase A (PKA) inhibitor, partially inhibited epinephrine stimulation of glucose transport, as opposed to the complete inhibition by ICI 118,551 (Figure 5D). The high mPKI concentration we used is known to cause complete inhibition of PKA in *ex vivo* preparations (Nalli et al., 2014). If mPKI is able to cause complete inhibition of PKA in the epithelial cells of the everted sacs, these results would suggest that also PKA-independent signaling is involved in epinephrine stimulation of glucose transport through the *β*_2_-AR.

Next, we investigated whether sugar sensing by apical *β*_2_-ARs could enhance glucose transport into the everted intestinal sacs. To avoid interference with glucose uptake by SGLT1 into the everted sacs, the *β*_2_-AR was stimulated with mannose. Mannose is transported by SGLT4 but is not a substrate of SGLT1 (Tazawa et al., 2005; Wright et al., 2011). Hence, any stimulation of glucose uptake through SGLT1 by mannose has to act through another target. We previously showed that mannose is also one of the sugars that potently stimulates the *β*_2_-AR, as measured in stably transfected Flp-In-293 cells incubated throughout in 5 mM glucose, and ICI 118,551 completely blocked the mannose response (Figure 5E). Five mM glucose or mannose equally potentiated the response of *β*_2_-ARs to 5 nM epinephrine, compared to sugar-free medium (to which 4 mM glutamine was added as energy source). Whereas 5 nM epinephrine still stimulated the *β*_2_-AR in sugar-free medium, although significantly less compared to medium containing 5 mM glucose or 5 mM mannose, mannose could only activate the *β*_2_-AR in cells pre-incubated with glucose or mannose, and not in sugar-free medium (Figure 5F). In everted sacs, both glucose transport and the stimulation of glucose transport by mannose were completely inhibited by LX4211, a dual inhibitor of SGLT1 and SGLT2 (Figure 5G). The stimulation of glucose transport by mannose was abolished by the *β*_2_-AR-antagonist ICI 118,551 (Figure 5H), indicating that mannose stimulates glucose transport through *β*_2_-ARs. Thus, sugar sensing by apical *β*_2_-ARs in enterocytes stimulates glucose absorption through SGLT1. ICI 118,551 (10 μM) by itself did not have any effect on glucose transport in everted sacs (Supporting Information Figure S10). In this experiment, the everted sacs were incubated in a lower glucose concentration of 2.5 mM compared to the earlier experiments with 5 mM glucose, to reduce activation of *β*_2_-ARs by glucose.

### STD-NMR demonstrates direct glucose binding to the *β*_2_-AR

**FIGURE 6:**
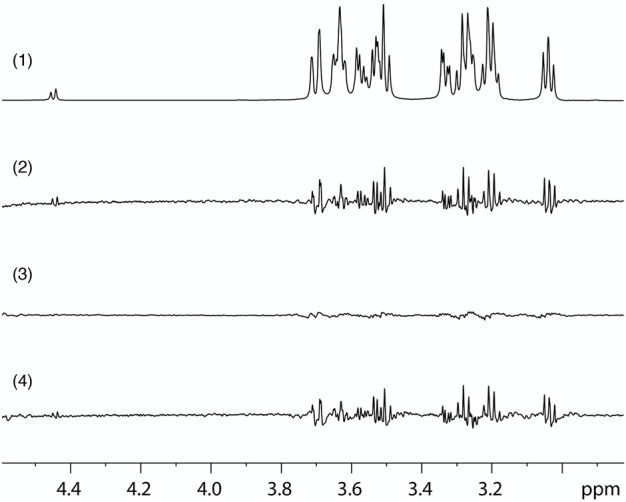
STD-NMR evidence for direct glucose binding to β2-AR. (1) Region of the 1D ^1^H NMR spectrum, obtained with a pure glucose solution, using with water suppression and showing the glucose signals. (2) STD spectrum on a sample containing glucose and a membrane preparation with high expression of *β*_2_-AR (Flp-In-293 cells) obtained by subtracting a spectrum with irradiation at .7 ppm, which saturates the receptor, from one with irradiation at 12 ppm, which does not affect the receptor. (3) STD control spectrum of a sample containing glucose and a membrane preparation without recombinant *β*_2_-AR (HEK Flp-In 293 cells), obtained with identical parameters as the spectrum shown in (2). (4) STDD spectrum obtained by subtraction of the STD spectrum of the control sample without *β*_2_-AR (3) from that of the sample containing *β*_2_-AR (2). The results show STD effects on the glucose ligand that arise from binding interaction with the *β*_2_-AR.

To confirm direct binding of glucose to the *β*_2_-AR, Saturation Transfer Difference (STD) NMR spectroscopy was performed with a membrane preparation containing the *β*_2_-AR, derived from the transfected Flp-in-293 cell line, and a control membrane preparation, with minimal inherent expression of the *β*_2_-AR, derived from the parent untransfected HEK Flp-In 293 cell line, similar to previous work showing direct binding of sugars to the human sweet taste receptor (Assadi-Porter et al., 2008; Assadi-Porter et al., 2010). STD NMR has proven to be a powerful technique to study ligand binding to membrane proteins (Mayer and Meyer, 1999; Venkitakrishnan et al., 2012). The STD spectrum of the *β*_2_-AR-containing membranes in the presence of 10 mM glucose was very pronounced, while the spectrum of membranes lacking *β*_2_-ARs but with the same glucose concentration had only a very small amplitude (Figure 6). The difference STDD spectrum provides clear evidence for direct binding of glucose to the *β*_2_-AR, since the saturation transfer can only take place upon physical binding of glucose to the target, the *β*_2_-AR, which is the only different component between sample and control membranes. This suggests that glucose interaction is not restricted to apical *β*_2_-ARs in intestinal epithelial cells, but that *β*_2_-ARs in general can interact with glucose and thus may act as a glucose sensor (or at least respond to glucose) also elsewhere in the body.

### Inhibition of apical *β*_2_-AR in gut epithelium with a non-bioavailable β-antagonist reduces blood glucose levels after an oral glucose bolus in rats

**FIGURE 7:**
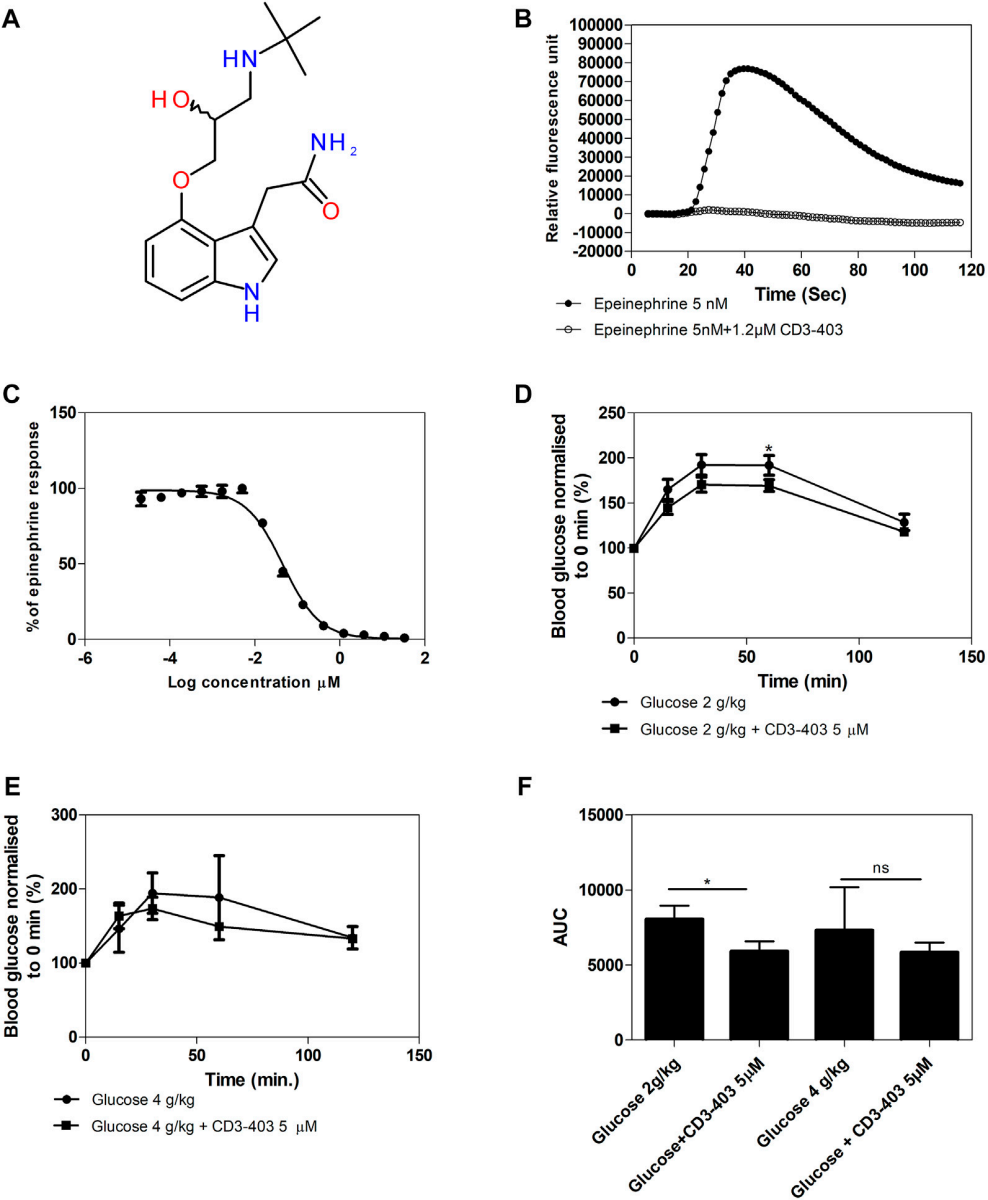
Blood glucose levels after administration of glucose to rats in the presence and absence the non-bioavailable *β*-AR antagonist CD3-403. **(A)** Structure of the non-bioavailable *β*-AR antagonist CD3-403, a modified, more polar derivative of ICI 118,551. **(B)** CD3-403 inhibits epinephrine-elicited *β*_2_-AR-mediated calcium response *in vitro* in Flp-In-293 cells. *X*-axis shows time in s, and *y*-axis displays fluorescence intensities (arbitrary units) for 5 nM epinephrine and 5 nM epinephrine +1.2 µM CD3-403 (representative graph from *n* = 3). **(C)** Concentration response curve for CD3-403 inhibition in Flp-In-293 cells, IC_50_ = 46 ± 5 nM. *X*-axis shows log concentration CD3-403 (µM) and *y*-axis displays fluorescence intensities (arbitrary units), normalized to epinephrine response, which was set at 100% (*n* = 3). **(D,E)** A bolus of glucose 2 g/kg body weight (4 sets of 3 rats), **(D)** or 4 g/kg body weight (1 set of 3 rats), **(E)** was administered orally to sets of rats in the absence or presence of 5 µM (.05 mg/kg) CD3-403. Glucose levels were measured in blood drawn from the tail vein at different time points after oral administration of the glucose bolus. The increase in blood glucose concentration is shown after the glucose load, normalized to the 0 min blood glucose concentration of 82 mg/dl for the whole group of 2 g/kg body weight and 85 mg/dl for the whole group of 4 g/kg body weight with or without CD3-403 treatment, which was set at 100%. (**F)**, For the experiment with glucose 2 g/kg, the area under the curve (AUC in units of blood glucose %. min) was calculated by setting 100% as a baseline. All values are expressed as mean ± SEM, *p* < .05: *. In case of the *in vivo* glucose bolus administration, the direction of the effect that we wanted to test was known, i.e. inhibition by CD3-403, hence a one tailed *t*-test was applied.

Finally, we have performed *in vivo* experiments in which rats were orally administered a glucose bolus together with a non-bioavailable β-antagonist: CD3-403 (Figure 7A). This compound was specially designed for our work based on the structure of ICI 118,551, which was modified by addition of a hydrophilic group and elimination of a hydrophobic group to ensure minimal permeability through biological membranes. This was confirmed by a Caco-2 permeability assay (Supporting Information Figure S2). CD3-403 inhibited with an IC_50_ of 46 nM the calcium response elicited by 5 nM epinephrine in Flp-In-293 cells stably transfected with *ADRB2* and *GNA15* (Figures 7B,C)*.* The CD3-403 compound was synthesized at 100 mg scale for oral administration in rats (GVK Biosciences Private Limited). The glucose bolus (2 g/kg or 4 g/kg body weight) was administered by oral gavage to sets of three rats each, in the absence or presence of 5 µM (.05 mg/kg) CD3-403. Glucose levels were measured in blood withdrawn from the tail vein just before, as well as 15, 30, 60, and 120 min after oral administration of the glucose bolus. The increase in blood glucose concentration after the glucose load is shown, normalized to the 0 min blood glucose concentration, which was set at 100%. In the case of glucose 2 g/kg, the presence of the CD3-403 compound caused a reduced increase in blood glucose level, which was significant at 60 min (Figure 7D). For the experiment with glucose 4 g/kg, we also saw a trend of decreased glucose concentration after treatment with CD3-403 (Figure 7E). For the experiment with glucose 2 g/kg, the area under the curve (AUC) was also calculated by setting 100% as a baseline. The CD3-403 compound significantly reduced the glucose increase (5,907 ± 2,195; mean ± SD) compared to the glucose 2 g/kg control group (8,056 ± 2,990; mean ± SD) (Figure 7F).

## Discussion

### The *β*_2_-AR has an additional function in glucose sensing

Our work provides strong evidence for an additional function of the *β*_2_-AR as glucose receptor. Although the different results obtained might each be subject individually to more or less likely alternative explanations, the comprehensive body of evidence when taken together makes a compelling and consistent case that *β*_2_-ARs in the apical enterocyte membrane sense the level of glucose in the gut to stimulate its uptake through SGLT1. This additional function is unexpected because the *β*_2_-AR is one of the best-characterized GPCRs. It has served as a leading model for elucidation of the mechanisms involved in GPCR signaling, the three-dimensional structure of GPCRs and the conformational changes triggered by ligand binding (Cherezov et al., 2007; Rasmussen et al., 2007; Rosenbaum et al., 2007; Shukla et al., 2008; Rasmussen et al., 2011a; Rasmussen et al., 2011b). As a major drug target, its pharmacology has been studied in great detail (Smith and Teitler, 1999; Baker, 2005; Frishman, 2008). It is the strong expression of *β*_2_-ARs in the apical plasma membrane of the epithelial cells facing the lumen of the gut, a highly unusual location for a receptor sensing a hormone distributed through the bloodstream, that led us to the current discovery. Interestingly, strong expression of *β*_2_-ARs in the apical membrane of the epithelium lining the proximal tubule of the nephron had already been reported (Boivin et al., 2001), as well as stimulation of glucose reabsorption from the kidney tubules by epinephrine (Blake, 1962). This apical location was interpreted, however, to allow for a response to epinephrine leaking from the blood into the urine primary filtrate. Similarly, *β*_2_-AR in the apical membrane of enterocytes might be activated fortuitously by epinephrine leaking through the tight junctions of the gut epithelium, although this is likely limited to pathological conditions given the lack of permeability in the tight junctions for small molecules like sugars and amino acids (Goff, 2018). Moreover, such explanations do not contradict our results demonstrating that enterocyte apical *β*_2_-AR functions as a glucose receptor for stimulation of sugar uptake from the gut. Multiple studies on intestinal epithelial cells have reported results consistent with those in our work. Expression of *β*-AR subtypes in the gut has been demonstrated by [^125^I]cyanopindolol binding and its competition with adrenoreceptor antagonists (Yu and Ouyang, 1997). mRNA expression and apical membrane location of *β*_2_-ARs was reported in a study on the regulation of apically located CFTR in murine duodenal epithelia (Singh et al., 2009). Moreover, *β*_2_-AR agonists activate CFTR in intestinal organoids through cAMP signaling (Vijftigschild et al., 2016), and optimal activity of CFTR in Caco-2 human colon carcinoma cells is dependent on the presence of glucose (Mailleau et al., 1998). Coupling of the *β*_*2*_ *-*AR to adenylate cyclase has been demonstrated in Caco-2 cell membranes based on the increase in cAMP level upon addition of different agonists (Re et al., 2001). Evidence was reported for an electrogenic response mediated by *β*_2_-ARs located in the basolateral membrane of distal colonic brush border basolateral membranes mounted in Ussing chambers (Zhang et al., 2009). Since the primary nutrients available in the colon are short-chain fatty acids produced by bacterial fermentation, rather than glucose, the *β*_2_-AR localization pattern may be specific for colonic epithelial cells. Moreover, the presence of *β*_2_-ARs on the basolateral membrane does not have to be in contradiction with our demonstration that (the vast majority of) the *β*_2_-AR is expressed on the apical membrane. The former makes physiological sense in the epinephrine-induced flight or fight response, in which the blood glucose level is increased to provide more glucose to the body. Hence, apical SGLT1 activity in the enterocytes could be enhanced by two mechanisms: epinephrine-induced stimulation from the serosal side through basolateral ß_2_-ARs and glucose-induced stimulation from the gut luminal side through apical ß_2_-ARs.

### Comparison with *β*_2_-ARs in the apical membrane of kidney tubule cells

The SGLT2, and to a lesser extent SGLT1, present in the apical membrane of the kidney tubule, are responsible for the reabsorption of glucose from the urine primary filtrate (Wright et al., 1997). Their expression is enhanced by luminal glucose (Vestri et al., 2001) and their plasma membrane insertion and/or activity are enhanced by elevated cAMP-PKA signaling, as in intestinal (Wright et al., 1997; Shepard and Pluznick, 2017) and ovary (Subramanian et al., 2009) epithelial cells. Similar regulation of intracellular SGLT1 and SGLT2 trafficking to the plasma membrane both in small intestinal mucosa and kidney tubules by RSCIA1 (RS1) has also been reported (Shepard and Pluznick, 2017; Schafer et al., 2019). Our results strongly suggest that *β*_2_-ARs may sense glucose also in kidney tubule epithelium to stimulate glucose recovery from the urine primary filtrate. Such a role makes more physiological sense than regulation by leaked catecholamines in the primary filtrate.

### Stimulation of glucose uptake by epinephrine in other cell types

Although the main acute effect of *β*_2_-AR stimulation is an increase in blood glucose concentration due to glycogen mobilization in the liver and inhibition of glucose disposal by insulin-dependent tissues, there have been many reports on stimulation of glucose uptake in different tissues by activation of adrenergic receptors independently of insulin (Abe et al., 1993; Liu and Stock, 1995; Faintrenie and Geloen, 1998; Doenst and Taegtmeyer, 1999; Nevzorova et al., 2002; Chernogubova et al., 2004; Hutchinson et al., 2005; Nevzorova et al., 2006; Kanda and Watanabe, 2007; Ngala et al., 2008; Catus et al., 2011). In astrocytes, adrenergic stimulation of the *β*_2_-AR acts through GLUT1 by coupling to Gs and activation of the adenylate cyclase pathway (Dong et al., 2012), while in rat skeletal muscle cells it causes increased translocation of GLUT4 to the plasma membrane (Nevzorova et al., 2002; Dehvari et al., 2012; Mukaida et al., 2019). Our results show that stimulation of glucose uptake through activation of *β*_2_-ARs does not only appear to target GLUT facilitated diffusion carriers in diverse somatic cell types, but also targets the active glucose transporter SGLT1 in intestinal epithelial cells. Involvement of the cAMP-PKA signaling pathway may be a common theme in adrenergic stimulation of glucose uptake.

### Connection with glucose sensing by the sweet taste receptors in the gut

It has been reported that knock-out mice in the sweet taste receptor T1R2 + T1R3 or in gustducin lack the secretion of gut hormones triggered by dietary sugars and also lack sugar-induced upregulation of SGLT1 expression and glucose absorptive capacity (Margolskee et al., 2007; Shirazi-Beechey et al., 2011a; Shirazi-Beechey et al., 2011b), while intestinal sweet taste receptor stimulation upregulates SGLT1 (Stearns et al., 2010). This sweet taste sensing system is expressed in enteroendocrine cells, and has to communicate with the enterocytes in which SGLT1 mediates bulk sugar uptake. Its inactivation apparently also disables the *β*_2_-AR-based sugar-sensing system in the enterocytes identified in this paper. An explanation may be that the enteroendocrine cells regulate the sensitivity of the *β*_2_-AR-based sugar-sensing system in the enterocytes, particularly over the longer term (hours to days). Inactivation of the enteroendocrine sweet taste sensing system may thus make the *β*_2_-AR sugar-sensing system in some way insensitive. This could happen at different levels, such as sorting of SGLT1 to the apical membrane and/or post-translational modification of SGLT1 or any component in the signaling pathway from *β*_2_-AR to the SGLT1 sorting mechanism. The increase of SGLT1-mediated glucose transport is based on different components with a divergent time-dependency, whose regulation may well be distinct (Gromova et al., 2021). Within seconds after exposure to glucose, pre-existing SGLT1 are translocated from a cytoplasmic pool to the cell surface membrane of the enterocytes. Those surface transporters then accelerate their glucose transport activity, presumably due to phosphorylation as reported for SGLT1 in Chinese hamster ovary cells (Subramanian et al., 2009). Later, synthesis of new SGLT1 protein is induced. Finally, the basal SGLT1 expression level may change over days or weeks depending on the food composition, as has been observed when herbivores are weaned and switch from a milk to a grass diet (Diamond and Karasov, 1987; Dyer et al., 2003). Our study has focused on the short-term SGLT1 changes, whereas many studies of the enteroendocrine pathways have focused on longer-term effects.

### Glucose sensing and the evolutionary origin of the *β*_2_-AR

GPCRs must be derived in evolution from proteins in unicellular organisms that lack the elaborate, extracellular endocrine signaling pathways present in higher eukaryotes, and nutrient-sensing GPCRs are prime candidates in this respect (Thevelein and Voordeckers, 2009; Nordstrom et al., 2011; Krishnan et al., 2012; de Mendoza et al., 2014). Although members of the main GPCR families have been found in the most primitive metazoan organisms (Nordstrom et al., 2011), the very poor sequence conservation between GPCRs makes it difficult to connect fungal and animal GPCRs in evolution (Krishnan et al., 2012). Our results suggest that *β*_2_-ARs evolved from an ancient glucose receptor, an ancestral GPCR present in a primitive unicellular eukaryote, similar to the Gpr1 glucose-sensing GPCR present in yeast and other fungi (Kraakman et al., 1999; Lemaire et al., 2004; Xue et al., 2008). Since sequence conservation between GPCRs is very limited in general, and especially between distant relatives like the yeast and mammalian GPCRs (Graul and Sadee, 2001), it is not possible at this point to make a meaningful prediction of a putative common glucose binding site. *β*_2_-AR immunoreactivity is abundant in the pharynx rim of the unicellular protozoon *Paramecium,* and was proposed to function as a nutrient sensor (Wiejak et al., 2002). It undergoes isoproterenol-induced desensitization by endocytosis, possibly initiated through phosphorylation by a homolog of the human β-adrenergic receptor kinase (βARK2, GRK3) (Wiejak et al., 2004). Expression of a *β*_2_-AR homolog in *Paramecium* was confirmed by RT-PCR and Northern blot analysis (Platek et al., 1999), and several adrenergic receptor orthologous genes have been annotated as such in the *Paramecium* genome (https://paramecium.i2bc.paris-saclay.fr/cgi/tool/search). Nascent phagosomes are formed at the pharynx rim, and β–adrenergic agonists stimulate phagocytosis in *Paramecium*, a response blocked by β–AR antagonists, and potentiated by forskolin, an activator of adenylate cyclase (Wyroba, 1987, 1989). Our results suggest that the *Paramecium β*_2_-AR homolog may indeed function as a sugar sensor, consistent with its localization at the pharynx rim and its involvement in triggering phagocytosis. Radioligand binding studies also provided evidence for the presence of β–ARs in the unicellular protozoon *Trypanosoma cruzi* (de Castro and Oliveira, 1987) and DNA hybridization for the presence of a *β*_2_-AR homolog in the slime mold *Dictyostelium discoideum* (Palacios et al., 1989).

### The binding site for glucose on the *β*_2_-AR

The STD-NMR results provide clear molecular evidence that glucose directly binds to the *β*_2_-AR, since the saturation transfer can only take place upon physical binding. The lack of competition between glucose and cyanopindolol for binding to the *β*_2_-AR (Figure 3A; Supporting Information Figure S5A) suggests that the binding sites are different. On the other hand, the inhibition of the glucose-induced response by classical *β*_2_-AR antagonists (Figure 2B) and the stimulation of cyanopindolol binding to the *β*_2_-AR by glucose (Figure 3A; Supporting Information Figure S5A), indicates that binding of a molecule into one of the two binding sites affects the other binding site and that therefore the two binding sites might be in close interaction and possibly close proximity. Antagonist binding into the epinephrine binding site might also compromise conformational changes involved in signaling from the glucose binding site. Glucose and epinephrine may bind into two subpockets of the orthosteric binding site, as recently reported for the binding of D-glucose and L-glucose in the sweet taste receptor TAS1R2/TAS1R3, which is also a GPCR (Dubovski et al., 2022). Further analysis of sugar binding affinity and specificity with purified *β*_2_-ARs and determination of the precise binding site in relation to that of *β*_2_-AR agonists and antagonists, goes beyond the scope of the present work, but should be a major focus in future research. This should also include the precise interaction of *β*_2_-AR with Gs proteins in response to glucose and epinephrine, as well as the possible sugar-sensing function of the other members of the *β*-AR family.

### Glucose sensing by *β*_2_-ARs in the gut

In the glucose-sensing yeast GPCR, Gpr1, evidence was reported for direct interaction of glucose with TMD VI (Lemaire et al., 2004), a transmembrane domain involved in binding of small ligand molecules in many GPCRs, including the *β*_2_-AR (Ghanouni et al., 2001). During its evolutionary development into a hormone receptor, the *β*_2_-AR has apparently retained its ancient glucose-sensing function. The unexpected localization of *β*_2_-ARs in the apical membrane of intestinal epithelial cells, making little physiological sense for a hormone receptor, has enabled us to identify this function. The *β*_2_-AR is used in enterocytes to sense luminal sugar and regulate its uptake from the gut. Interestingly, both the *β*_2_-AR and yeast Gpr1 use the cAMP-PKA signaling pathway to control storage carbohydrate levels and sugar catabolism (Thevelein and de Winde, 1999). Our results also explain previous observations that perfusion of rat small intestine with epinephrine significantly increases transport of glucose from the gut lumen to the blood (Aulsebrook, 1965; Ishikawa et al., 1997). This is associated with an increase in SGLT1 protein and phlorizin binding, and was also elicited by perfusion with dibutyryl-cAMP (Ishikawa et al., 1997). Epinephrine is a water-soluble compound with a very low membrane permeability coefficient of 2.7 ± 1.5 × 10^–6^ cm/s (Bochain et al., 1981), which precludes any significant passive diffusion through membranes. Hence, epinephrine should in principle not be able to reach the baso-lateral membrane of the epithelial cells when administered in the gut lumen. Aschenbach et al. (2002) reported stimulation of SGLT1-mediated glucose uptake in isolated ovine ruminal epithelia by several *β*_2_-AR agonists. Stimulation by forskolin, an activator of adenylate cyclase, and inhibition by the PKA inhibitor H89 supported involvement of cAMP-PKA signaling. These observations are consistent with the presence of *β*_2_-ARs in the apical membrane, and cAMP signaling as mediator of enhanced SGLT1 expression and glucose uptake as a result of *β*_2_-AR stimulation. Our discovery that the *β*_2_-AR can function as a sugar receptor on the apical side of the intestinal epithelial cells provides a logical explanation for upregulation of SGLT1 and glucose uptake elicited by perfused epinephrine. Future research should study in more detail the molecular mechanisms involved in *β*_2_-AR-mediated upregulation of SGLT1 at the transcriptional and post-translational level, including SGLT1 intracellular trafficking and ligand-induced endocytosis, as well as the composition of the signaling pathway(s) involved and their possible interaction with incretin hormones, and other mechanisms of neuroendocrine signaling.

### Gut glucose sensing by *β*_2_-ARs in knockout mice

It would be useful in principle to complement the present work with genetic experiments in mice lacking the *β*_2_-AR and testing for the rate of glucose transport from the gut to the blood. However, given the complex role of the *β*_2_-AR in glucose homeostasis in the mammalian body, acting in different ways on glucose mobilization from reserve tissues and glucose sequestration in other tissues, it would be imperative to perform such genetic experiments with tissue-specific knock-out mice lacking the *β*_2_-AR specifically in the intestinal epithelium. Otherwise, interpretation of the results would likely be very cumbersome and inconclusive. On the other hand, we would like to emphasize that the use of a non-bioavailable beta-blocker to specifically inhibit the sensing of glucose by the *β*_2_-AR in the gut epithelium is a much stronger and more reliable scientific argument than the use of a tissue-specific knock-out because of the many possible complications that the latter may cause, including upregulation of related adrenergic receptors, abolishment of regular *β*_2_-AR interactions and possible adverse effects on intestinal epithelial cell development, as well as the general shortcoming that elimination of a physiological response by deletion of a receptor protein does not necessarily imply that the sensing function of the receptor is directly responsible for the absence of the response. Even with intestinal epithelial specific knock-out mice, a major question will be raised in case a reduction in the postprandial increase in blood glucose level is also observed. A control experiment will be needed to demonstrate that these *β*_2_-AR knock-out mice, which are defective from birth, display the same SGLT1-mediated basal glucose uptake activity in the intestinal epithelial cells. This question can only be answered with the pharmacological approach that we have used in our work using a non-bioavailable beta-blocker, that is only administered together with the glucose bolus and therefore minimizes the risk of affecting the basal activity of SGLT1 and/or any other component possible affecting the transepithelial uptake of glucose from the gut into the bloodstream.

### Post-translational modification of the *β*_2_-AR in the enterocyte apical membrane

Another aspect that is likely different between *β*_2_-ARs in the apical membrane of enterocytes compared to the plasma membrane of other cell types is the degree and nature of post-translational modification, in particular glycosylation. The extracellular domains of apical membrane proteins in enterocytes are uniquely and heavily glycosylated (Taatjes and Roth, 1991). This glycosylation changes during their differentiation as they migrate upwards from the crypt base to the villus tip, and it is also influenced by the composition of the gut content (Biol et al., 1992). Glycosylation often affects protein functionality (Moremen et al., 2012) and future research thus will have to determine whether changes in *β*_2_-AR glycosylation affect the affinity and/or specificity of the sugar-sensing function of apical *β*_2_-AR in the enterocytes.

### Therapeutic potential of enterocyte apical *β*_2_-ARs as drug target for diabetes and obesity

Reduction of glucose uptake from the gut by SGLT1 inhibitors is being explored for treatment of diabetes and obesity (Wagman and Nuss, 2001; Asano et al., 2004). Enhanced uptake of glucose from the gut by upregulation of SGLT1-activity was shown to cause obesity in mice (Osswald et al., 2005). The expression of SGLT1 and other sugar transporters was found to be upregulated in gut epithelium from diabetic patients, suggesting that an increased capacity to absorb sugars from the gut may reinforce diabetic syndromes (Dyer et al., 2002; Shepard and Pluznick, 2017; Koepsell, 2020). Similar findings were made for obese patients (Nguyen et al., 2015). Apical *β*_2_-ARs in enterocytes may constitute a more attractive target than SGLT1 for partially blocking postprandial glucose uptake from the gut in diabetic and obese patients, since antagonism of these *β*_2_-ARs only blocks the sugar-induced stimulation of glucose uptake and not total glucose uptake, as is the case with SGLT1 antagonists. The latter easily results in osmotic diarrhea, enhanced microbial activity and flatulence, as a consequence of excessive gut sugar levels (Soergel, 1994; Wright et al., 2007). Obviously, *β*_2_-AR antagonists with limited oral bio-availability would be the drugs of choice for this purpose, so as to avoid interference with the *β*_2_-AR in other tissues of the body. Our work has now shown that a non-bioavailable *β*_2_-AR antagonist is able to reduce the increase in blood glucose level when administered together with an oral glucose bolus. This suggests that non-bioavailable *β*_2_-AR antagonists could be useful in humans to lower the postprandial increase in blood glucose level, and therefore might be used to reduce glucose uptake in diabetic and obese patients. It has been reported previously that in an intraperitoneal glucose tolerance test in *β*_2_-AR knock-out mice (Santulli et al., 2012) or after introducing an intravenous blood glucose load together with a *β*_2_-AR antagonist in rats (Skikama and Ui, 1975) or diabetic patients (Holm et al., 1980), a much higher increase in blood glucose level was observed compared to controls. This supports our conclusion that the reduction in blood glucose level observed in our *in vivo* experiments cannot be due to systemic inhibition of *β*_2_-ARs, but must rather result from inhibition of the apical *β*_2_-ARs in the gut epithelium. Although the reduction in the peak blood glucose level was limited, it was significant and might be enough for long-term beneficial effects. Recent work has shown that limited lowering of the postprandial blood glucose level caused by the presence of polymorphisms in SGLT1 in specific human subpopulations led to long-term beneficial effects, reducing the incidence of diabetes, obesity, heart failure and mortality (Seidelmann et al., 2018). Future research should study the effect of administration of non-bioavailable *β*_2_-AR antagonists as well as agonists in different nutrient regimes on glucose homeostasis, body weight gain and levels of insulin, glucagon, ghrelin, GLP-1 and GIP incretins, and other hormones known to be linked to glucose homeostasis in the body, in healthy individuals as well as in diabetic and obese patients.

### Physiological role of glucose sensing by the enterocyte apical *β*_2_-AR in the gut

We have used experimental conditions that in our view would maximize the chance of detecting a significant effect on the blood glucose level by administration of a non-bioavailable *β*_2_-AR antagonist. However, it is unclear what the main driving force was in evolution to establish and maintain a glucose receptor in the gut to stimulate glucose uptake. Was it to maximize high glucose uptake during sparse meals? Or was its main function to stimulate uptake of low glucose levels under malnutrition conditions? Future research will have to investigate the effect of non-bioavailable *β*_2_-AR antagonists under a variety of feeding conditions, not only on blood glucose levels, but also on general glucose homeostasis and other relevant parameters, such as body weight gain. A possible connection with regulation of GLUT2 is also relevant in this respect since GLUT2 was shown to be translocated from the basolateral to the apical membrane of enterocytes at high (>30 mM) intestinal glucose levels (Kellett and Helliwell, 2000; Gromova et al., 2021). The co-transport of Na^+^ and glucose through SGLT1 was suggested to cause plasma membrane depolarization and cellular Ca^2+^ influx, resulting in increased translocation of GLUT2 to the apical membrane (Kellett et al., 2008). Future research should reveal whether the sensing of high intestinal glucose levels by *β*_2_-AR might also be involved in stimulating translocation of GLUT2 to the apical membrane.

### Possible functioning of the *β*_2_-AR as glucose receptor in other tissues

The *β*_2_-AR might function as a combined epinephrine/glucose receptor. Since glucose serves as essential carbon and energy source for virtually all cells of the body, this would provide an explanation why the *β*-ARs are expressed in virtually all tissues of the body in spite of classically considered to function in the very specific fight or flight response (Barnes, 1995; Insel, 1996; Mersmann, 1998). Glucose-induced regulatory effects are also quite common in mammalian tissues, which is not surprising in view of the crucial role of glucose as a nutrient throughout the body. It is presently unclear whether the *β*_2_-AR may also serve as glucose receptor for regulation of glucose-controlled processes in cell types or tissues other than those investigated in this study. The glucose-sensing function of the *β*_2_-AR might play a role in multiple ways in the complex regulatory network controlling glucose homeostasis in the body. For instance, glucose sensing by *β*_2_-ARs may modulate epinephrine sensing in blood and other body fluids, as suggested by the glucose stimulation of [^125^I]cyanopindolol binding to the *β*_2_-AR (Figure 3A, Supporting Information Figure S5A), or may support feedback inhibition of *β*_2_-ARs by high glucose levels through desensitization, stimulation of its endocytosis and/or prevention of its recycling (Moore et al., 2007; Yudowski et al., 2009).

### Glucose sensing by *β*_2_-ARs may explain hitherto unexplained pathophysiological effects

The glucose-sensing function of the *β*_2_-AR may provide an explanation for some of the hitherto unexplained observations in human (patho)physiology related to *β*_2_-AR function, such as unexpected negative side effects of *β*-AR antagonist therapy (Sears, 2002; Barron et al., 2013) or unexplained differences in therapeutic outcomes between different *β*-AR antagonists (Messerli and Grossman, 2004; Leonetti and Egan, 2012). The abnormally high glucose levels in diabetic patients may cause spurious activation (or desensitization) of *β*_2_-ARs throughout the body, and be responsible for hitherto unexplained symptoms and complications of diabetes, like the well-established correlation between diabetes, hypertension and heart failure (Nilsson et al., 2011). Deleterious consequences of prolonged overstimulation of beta-adrenergic receptors have been amply documented, for instance in heart failure (Taylor, 2007). Although epinephrine is well known to increase blood glucose levels through stimulation of glycogen breakdown, this appears to be a short-lived effect. In the long term, epinephrine increases muscle glucose uptake (Elayan et al., 2012; Ziegler et al., 2012). Stimulation of glucose uptake by epinephrine has been documented in muscle upon chronic administration (Jensen et al., 2005) and also in brown adipose tissue (Ernande et al., 2016). Stimulation of glucose uptake by *β*_2_-ARs may be relevant in other cell types as well. Particularly cells in which very active glucose uptake is critical, e.g. in cancer cells where the reasons for the strong correlation between *β*_2_-AR expression and cancer aggressiveness (Cole and Sood, 2012), the frequent involvement of *β*_2_-ARs in multiple carcinogenic processes (Perez-Sayans et al., 2012), as well as the beneficial effect of *β*-AR antagonists on the recovery of cancer patients during chemotherapy (Ji et al., 2012), have remained enigmatic up to now. The *β*_2_-AR has also been reported to show basal ligand-independent activity, whereas this property is considerably weaker in the closely related *β*_1_-AR subtype (Chakir et al., 2003; Rasmussen et al., 2007). Since glucose is present in most experimental media and body fluids, this sugar may have contributed to the basal “ligand-independent” activity, particularly since ICI 118,551 (which blocks the *β*_2_-AR response to glucose) can block this spontaneous activity (Chakir et al., 2003).

## Conclusion

In conclusion, we have discovered that the *β*_2_-AR, a well-established catecholamine receptor, is located at the apical side of intestinal epithelial cells to serve as a sugar sensor for stimulation of glucose uptake by SGLT1 from the lumen of the mammalian gut. We demonstrate direct binding of glucose at physiological concentrations to recombinant *β*_2_-ARs contained in membrane vesicles, suggesting that the *β*_2_-AR may be able to exert a more general glucose-sensing function also in other cells and tissues, and in other organisms.

## Data Availability

The original contributions presented in the study are included in the article/supplementary material, further inquiries can be directed to the corresponding author.
