# Supplementary material for: The β2-adrenergic receptor in the apical membrane of intestinal enterocytes senses sugars to stimulate glucose uptake from the gut
Source: Front Cell Dev Biol. 2023 Jan 9;10:1041930. doi: 10.3389/fcell.2022.1041930 (PMC9869975; doi:10.3389/fcell.2022.1041930)
Supplement: Supplementary file 1 [file DataSheet1.PDF]

Supporting Information

Table of contents

Supporting Information Figures

Figure 1

Figure 2

Figure 3

Figure 4

Figure 5

Figure 6

Figure 7

Figure 8

Figure 9

Figure 10

Supporting Information Tables

Table 1

Table 2

a

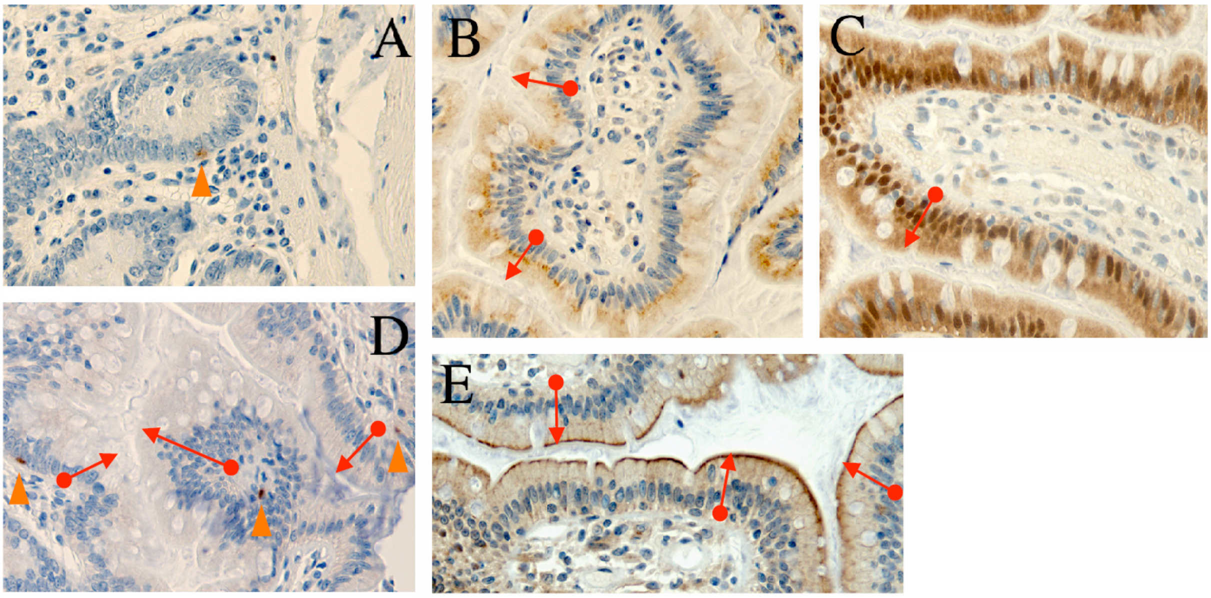

b

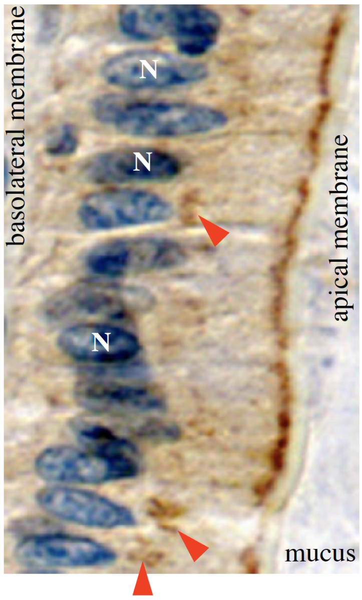

Supporting Information Figure 1

Immunohistochemistry of human biopsy samples from villi of the small intestine. **a,** immunoreactivity of selected GPCRs revealing different cellular and subcellular localizations. Orange arrowheads: immunoreactivity. Red arrows: epithelial orientation, with the basolateral/serosal side as the starting point and the arrow heads indicating the apical/luminal

side. A. anti-GPR105 antibody, B. anti-GPR1 antibody, C. anti-GPR120 antibody, D. anti-GPRC5C antibody, E. anti-β2AR antibody. **b,** larger magnification of enterocytes in biopsy sections stained with anti-β2AR antibody. The pronounced staining at the apical membrane is clearly visible. Red arrowheads indicate weaker and more diffusive staining of immunopositive intracellular structures, possibly the ER/Golgi. There is no discernible staining at the basolateral membrane. N: nucleus.

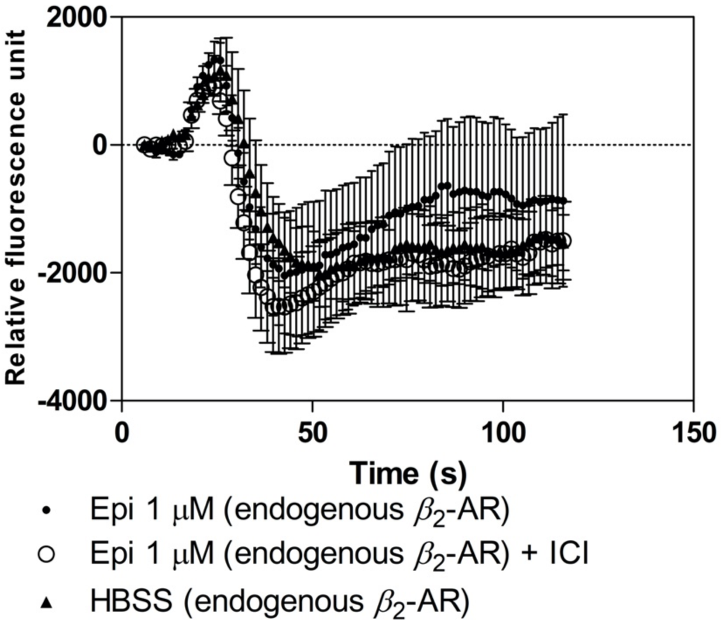

Supporting Information Figure 2

Effect of 1 μM epinephrine (Epi) and 1 μM epinephrine (Epi) together with 1 μM ICI 118,551 (ICI), a β_2-AR-specific inhibitor, on calcium response in HEK-293 cells. Epinephrine did not elicit a significant calcium response in HEK-293 cells via the endogenous β2-AR. X-axis shows time in s, and y-axis displays fluorescence intensity (arbitrary units) (n=3). All values are expressed as mean ± SEM.

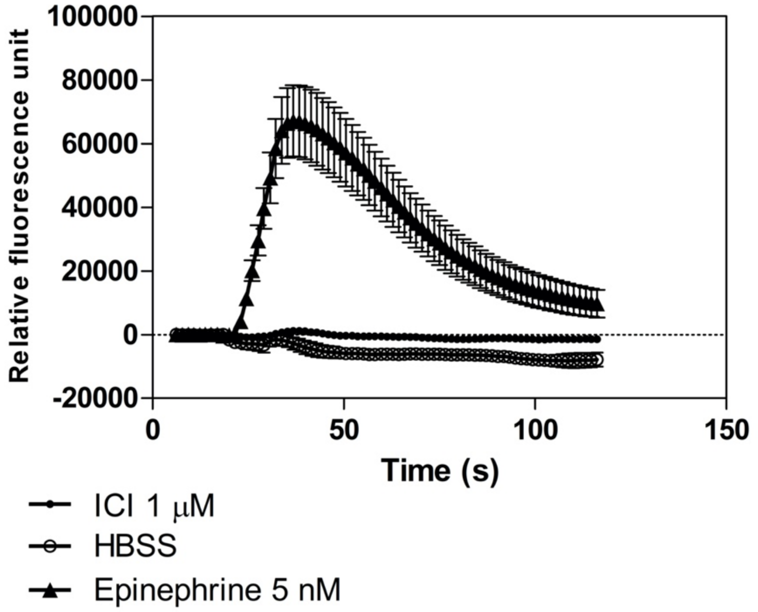

Supporting Information Figure 3

Effect of 1 μM ICI-118,551 (ICI), a β_2-AR-specific antagonist, in Flp-In-293 cells stably transfected with ADRB2 and GNA15. ICI-118,551 itself did not elicit a calcium response. Epinephrine (5 nM) was added as positive control, and Hank's Buffered Salt Solution (HBSS) as negative control. X-axis shows time in s and y-axis displays fluorescence intensity (arbitrary units) (n=3). All values are expressed as mean ± SEM.

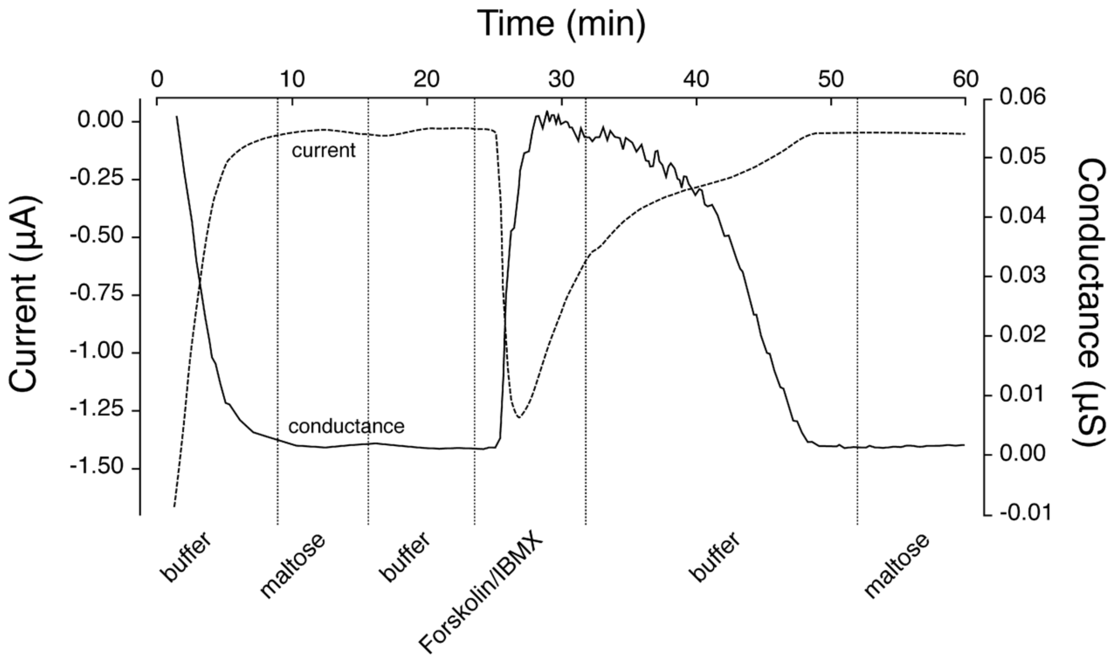

Supporting Information Figure 4

Absence of maltose response in *Xenopus* oocytes lacking heterologously expressed human β_2-AR. Forskolin/IBMX, which artificially increases the cAMP level, was added to demonstrate functionality of the CFTR channel. Addition of different glucose concentrations also had no effect on the membrane current and conductance in a non-injected oocyte.

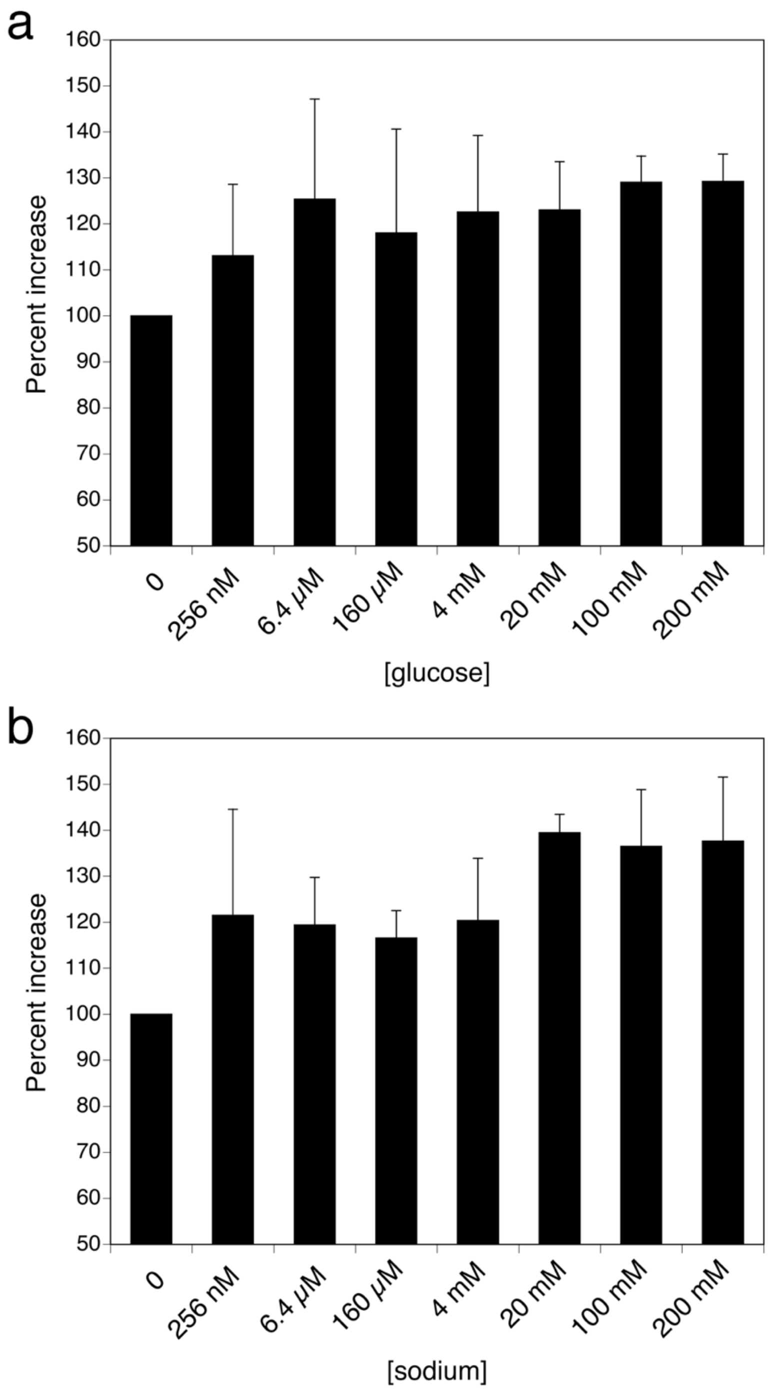

Supporting Information Figure 5

Binding (mean ± SEM) of ^{125}I-cyanopindolol to vesicles containing β_2-AR. **a,** Stimulation of the binding with increasing concentrations of glucose (in the absence of Na^+), **b,** Stimulation of the binding with increasing concentrations of Na^+ (in the absence of glucose). Note that the Y-axis starts at 50%. Binding in the absence of glucose (2000 – 2700 cpm) was normalized to 100%. Equal volumes of membrane suspension were used.

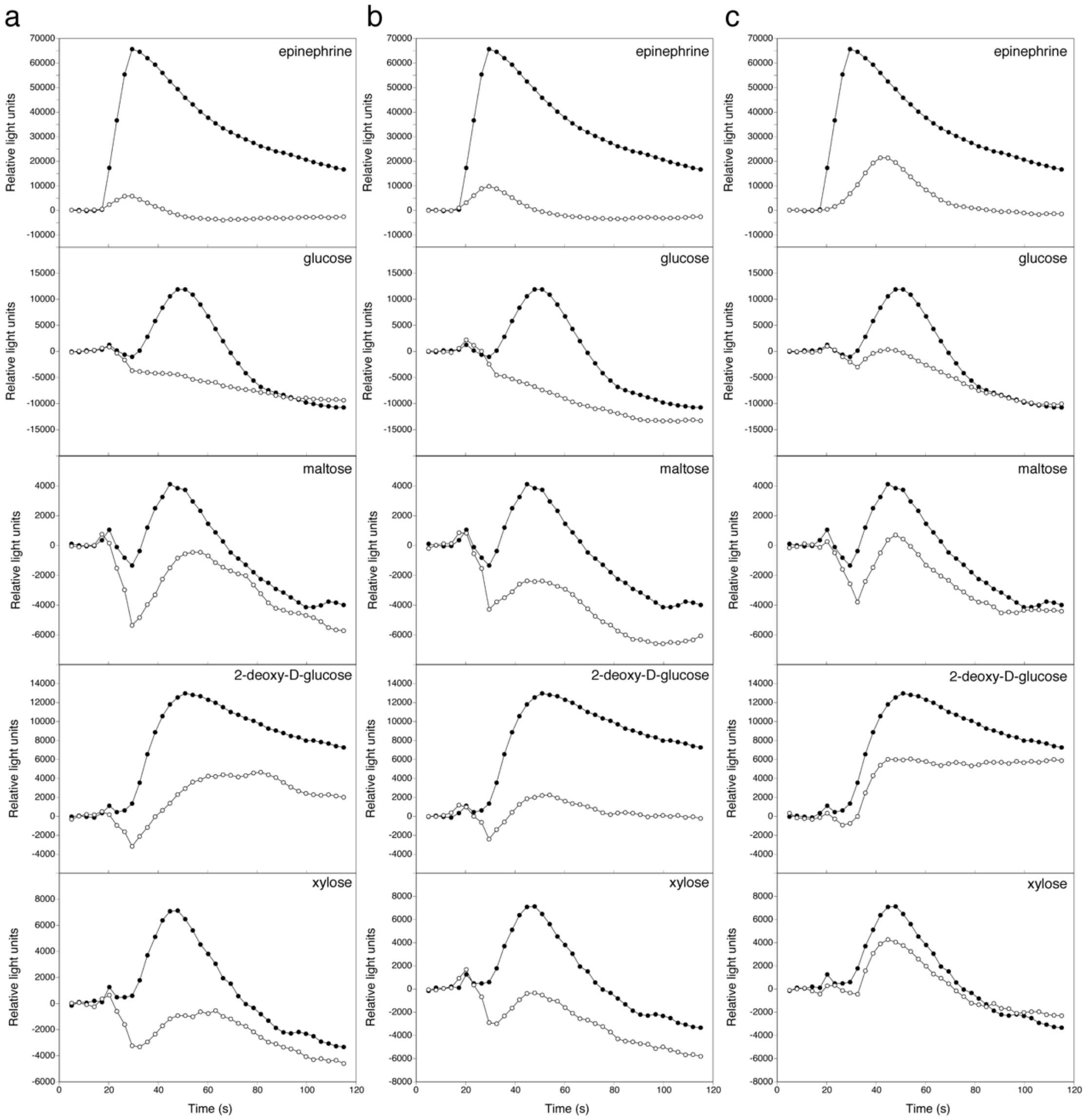

Supporting Information Figure 6

Inhibition of epinephrine and sugar responses with β_2-AR-specific antagonists. **a,** 200 μM propranolol, **b,** 200 μM labetalol, **c,** 200 μM nadolol. 20 nM epinephrine or 70 mM sugar was added in the absence (●) or presence of inhibitor (○).

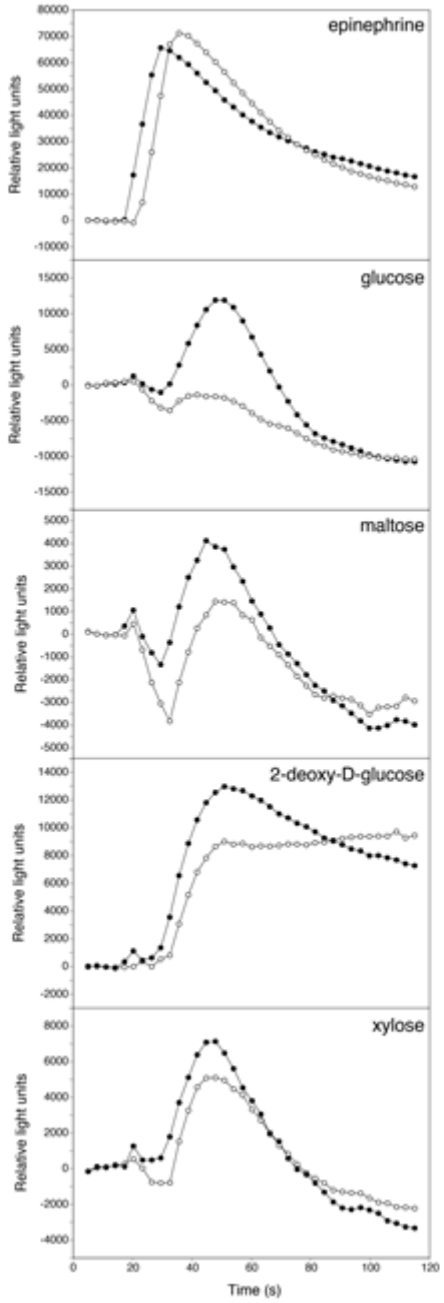

Supporting Information Figure 7

Differential inhibition of the sugar response with a β1-antagonist, metoprolol (200 μM); 20 nM epinephrine or 70 mM sugar was added in the absence (●) or presence (○) of antagonist.

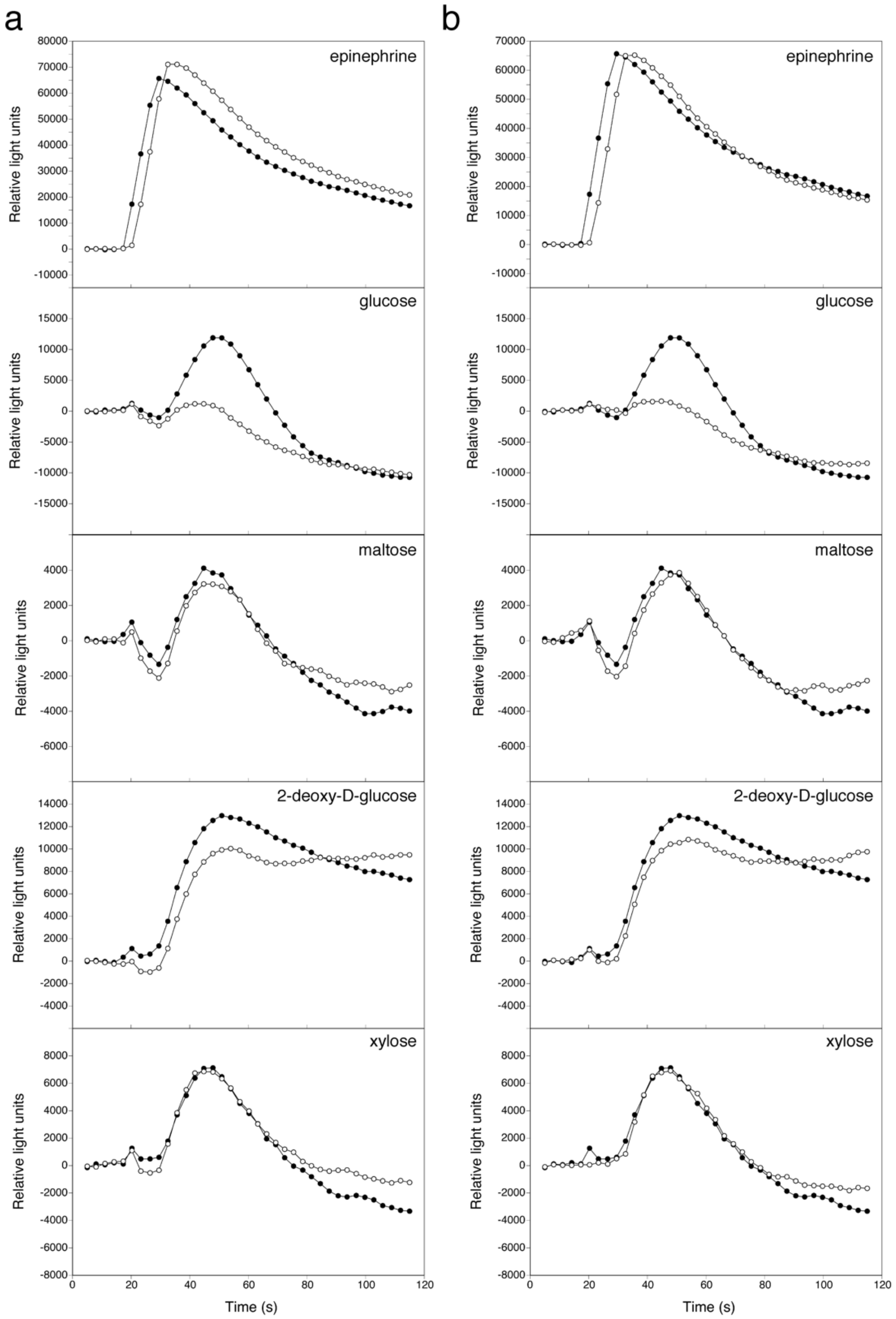

Supporting Information Figure 8

Absence of inhibition of the epinephrine response and differential inhibition of the sugar response with β1-antagonists. **a,** 200 μM acebutolol, **b,** 200 μM atenolol. 20 nM epinephrine or 70 mM sugar was added in the absence (●) or presence (○) of antagonist.

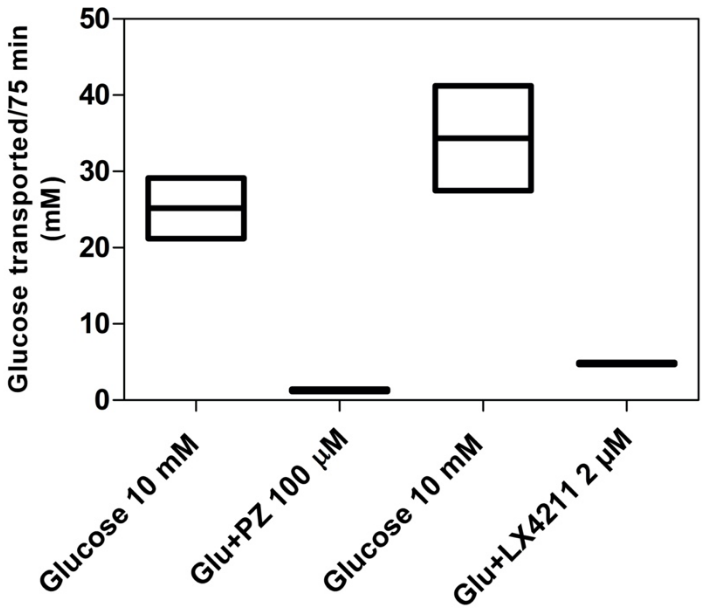

Supporting Information Figure 9

Effect of phlorizin (PZ) (100 µM) and LX4211 (2 µM) on glucose (Glu) accumulated after 75 min inside everted sacs, prepared from rat intestine. PZ and LX4211 inhibited more than 90 % of total glucose transport. (Respective number of replicates n=2, n=2, n=2 for conditions shown in the figure. For conditions with multiple measurements, the low and high values are indicated as the top and bottom of the box, with a horizontal line at the mean.)

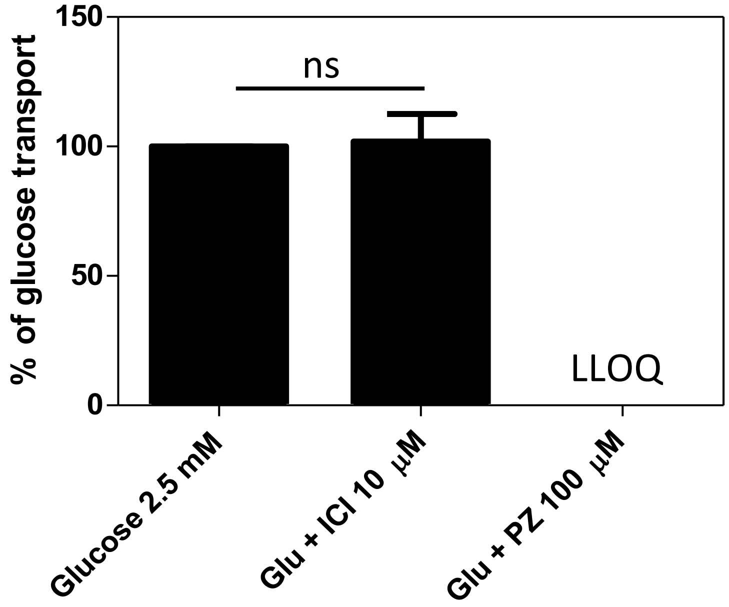

Supporting Information Figure 10

Effect of ICI-118,551 (ICI) and phlorizin (PZ) on glucose (Glu) accumulated after 10 min inside everted sacs, prepared from rat intestine. ICI itself did not have any effect on glucose transport. (Respective number of replicates n=6, n=7, n=3 for conditions shown in the figure; LLOQ is lower limit of quantification). The glucose transport for the different groups was expressed as a % of the glucose transport of the glucose control group. All values are expressed as mean ± SEM, $p > 0.05$: ns.

Supporting Information Table 1

PCR primers

Forward (fw) and reverse (re) primers used for reverse transcriptase PCR and for quantitative PCR (qPCR) were designed as required for SYBR Green (Eurogentec) qPCR reactions.

GPR1 fw TGGACCCCTTATCACCTGTTTAG

GPR1 re CCTGCAGCACATTCTGGAAA

GPR120 fw TCCGAGTGTCCCAACAAGACTA

GPR120 re ATGATGGGACTCCACATGATG

GPR125 fw CGTGCAGTTTCGAACAAACG

GPR125 re CTGGCCCGGTGTCCTTT

GPR18 fw GGAGAAGTCCATACGGATCATCA

GPR18 re GTGGAAGGGCACGAAGCA

GPR19 fw CTACCGCAGCAATGCCTACA

GPR19 re GATTTCCGAAATGCCCACAT

GPR22 fw TCGTGTTTGGTGTGAGAACTTCA

GPR22 re TCCCGGTGGCGTTTCA

GPR39 fw CCCATGGAGTTCTACAGCATCAT

GPR39 re CGTGTGGAGCTTACAGGACAGA

GPR40 fw TGCCTCCAATGTGGCTAGTTT

GPR40 re CCCTGTGATGAGTCCCAACTTC

GPR48 fw AAGACGACTGGAAGCTCCTGAA

GPR48 re CCGCCTTGGCTGCTGAT

GPR49 fw TGAGATCAAAACACGCGAGTCT

GPR49 re TGCTGGCGTGGGTAAAGG

GPR56 fw CCAAGGCTTCCTCATCTTCCT

GPR56 re GCGCTGTCTGAGTTGTTCTTCA

GPR61 fw AACCTGGATTGGCTACTTTTGC

GPR61 re TAAGCTCGCCCCGGATCT

GPR80 fw CCTTTGGCAACCTGCTGTTATAT

GPR80 re CCGCTGGCTTTGCATCTC

GPR85 fw TTGCACGAGGGCCTGTAGTAC

GPR85 re ATTCCTGCTTGGGCGAAACT

GPR88 fw GCTCTACACGTGGAGGAACGA

GPR88 re GTTGCGCCTGGGACATG

TM7SF1 fw CCCCCGAAGATATGACAGTGAT

TM7SF1 re GGAGCAAAACTTCCCTGAAGTC

TM7SF2 fw GCAGCAGTGCCTCCAAAAGT

TM7SF2 re GGTAAGGCACACGCTTGCA

TM7SF3 fw GAAAGAGGACAGCCGCCTTT

TM7SF3 re GATGTTGGTCACTCTGCGTTCTC

Supporting Information Table 2

Caco-2 cell permeability data for compound CD3-403, measured from apical to basal (A-B) and from basal to apical (B-A), at pH 7.4 in both the apical and basal compartment.

**Compound**	**Test Concentration**	**A-B permeability** (10^{-6} cm/s)	**Mean Recovery** (%)	**B-A permeability** (10^{-6} cm/s)	**Mean Recovery** (%)	**Efflux**
CD3-403	1.0 µM	0.4	83	1.3	94	3.25

Low permeability: < $2x10^{-6}$ cm/s
Medium permeability: $2x10^{-6}$ cm/s < permeability < $20x10^{-6}$ cm/s)
High permeability: > $20x10^{-6}$ cm/s
